# Joint association of TyG-ABSI composite index and inflammatory score with diabetes in middle-aged and older Chinese adults: a prospective cohort study from CHARLS (2011–2020)

**DOI:** 10.1186/s12902-026-02370-3

**Published:** 2026-06-19

**Authors:** Qingqing Liu, Kunjie Zheng, Liping Hou

**Affiliations:** Department of Endocrinology, Hengshui People’s Hospital, Hengshui, 053000 China

**Keywords:** TyG-ABSI, Inflammation, Type 2 diabetes, CHARLS

## Abstract

**Background:**

Diabetes mellitus represents a major global public health challenge, especially in middle-aged and older populations. The triglyceride-glucose A Body Shape Index (TyG-ABSI) composite index and inflammatory score are potential predictors of type 2 diabetes mellitus (T2DM). However, their independent and joint associations with incident T2DM in middle-aged and older Chinese adults remain unclear.

**Methods:**

Data were obtained from the China Health and Retirement Longitudinal Study (CHARLS, 2011–2020), including 6,216 participants without diabetes at baseline. The TyG-ABSI index and inflammatory score (constructed from high-sensitivity C-reactive protein and white blood cell counts) were calculated. Multivariate Cox regression models, restricted cubic splines were used to evaluate their associations with incident T2DM.

**Results:**

Higher TyG-ABSI and higher inflammatory score were independently associated with an increased T2DM risk (fully adjusted hazard ratio [HR]: 1.42, 95% confidence interval [CI]: 1.17–1.72 and 1.21, 95% CI: 1.02–1.44, respectively). Participants with concurrent high TyG-ABSI and high inflammatory score had a 69% higher T2DM risk (HR: 1.69, 95% CI: 1.30–2.21) than the reference group. Restricted cubic spline analyses showed approximately linear dose-response relationships. The combined index demonstrated superior discriminative ability (Harrell’s C-index) and significantly improved risk reclassification (NRI) and overall discrimination (IDI) compared with traditional risk factors alone.

**Conclusion:**

TyG-ABSI composite index and inflammatory score were independently and jointly associated with incident T2DM in middle-aged and older Chinese adults. This combined application can improve T2DM risk stratification and facilitate early prevention.

**Clinical trial number:**

Not applicable.

**Supplementary information:**

The online version contains supplementary material available at 10.1186/s12902-026-02370-3.

## Research insights

**Table:** 

Item	Content
What is currently known about this topic?	The TyG index reflects insulin resistance. The ABSI provides a better assessment of abdominal fat. Inflammation contributes to the development of type 2 diabetes.
What is the key research question?	Are TyG-ABSI and inflammatory score independently and jointly associated with incident type 2 diabetes in middle- aged and older Chinese adults?
What is new?	TyGABSI and inflammatory score independently predict diabetes. Their combination increases the risk by 69%, and a linear doseresponse relationship was observed.
How might this study influence clinical practice?	The combined index enables simple, cost-effective diabetes risk stratification for early prevention.

## Background

Diabetes mellitus remains a major global public health challenge. According to the 11th edition of the IDF Diabetes Atlas (2025), an estimated 589 million adults aged 20–79 years lived with diabetes worldwide in 2024, corresponding to a prevalence of 11.1%. China has the largest diabetes population globally, with approximately 148 million affected adults in the same age group and an age-standardized prevalence of 11.9% [1]. Type 2 diabetes mellitus (T2DM) is the most common form of diabetes [2]. Complications that have traditionally been associated with diabetes mellitus include macrovascular conditions such as coronary heart disease, stroke, and peripheral arterial disease, and microvascular conditions such as diabetic kidney disease, retinopathy, and peripheral neuropathy [3]. With advances in diabetes management, associations with cancer, infections, functional and cognitive disabilities, liver disease, and affective disorders are increasingly recognized [4]. These complications severely impair the patients’ quality of life and increase medical costs; therefore, early identification of high-risk groups and exploration of reliable predictive indicators are critical for strengthening T2DM prevention and control.

Insulin resistance is a fundamental pathophysiological mechanism underlying T2DM development [5]. The triglyceride-glucose (TyG) index, calculated from fasting triglyceride and glucose levels, has gained recognition as a reliable surrogate marker of insulin resistance [6, 7]. A longitudinal study spanning 12 years in non-obese adults confirmed the involvement of the TyG index in T2DM pathogenesis [8]. Extensive population-based studies have underscored the predictive ability of the TyG index for assessing T2DM risk [9, 10].

Visceral obesity, a key pathological basis of diabetes [11], impairs pancreatic β-cell function, disrupts insulin signaling to induce insulin resistance, and accelerates β-cell dysfunction via chronic inflammation and oxidative stress [12]. Obesity is usually described by the body mass index (BMI). However, BMI does not distinguish between muscle and fat accumulation, nor does it differentiate among distinct fat distribution patterns in the body. Krakauer et al. proposed the A Body Shape Index (ABSI) to overcome this limitation, which standardizes waist circumference (WC) by height and BMI. This index more accurately reflects abdominal fat distribution, independent of total body size [13]. ABSI shows a stronger independent association with mortality risk in older adults than BMI, serving as a valuable supplement to BMI in assessing the health status of this population [14]. Additionally, as an independent risk factor for incident diabetes, the ABSI exhibits a nonlinear dose-response relationship with diabetes risk and demonstrates moderate predictive efficacy for future diabetes onset [15].

To boost predictive accuracy, researchers have constructed a range of TyG-derived composite indices by integrating the TyG index with obesity-related parameters, encapsulating both insulin resistance and central obesity in a single metric. TyG-ABSI has demonstrated superior predictive value for cardiovascular disease, mortality, and stroke across diverse populations, including those with hyperuricemia, metabolic syndrome, and early-stage cardiovascular kidney metabolic syndrome, outperforming traditional TyG-derived indices and single anthropometric or metabolic markers [16–19]. However, the predictive value of TyG-ABSI for the incidence of diabetes has not yet been comprehensively assessed.

Inflammation plays a key role in the pathogenesis of T2DM [20], and elevated levels of inflammatory markers, including C-reactive protein (CRP) and white blood cell count (WBC), have been independently associated with increased diabetes risk [21–23]. Inflammatory scores are widely used to assess systemic inflammation and predict cardiometabolic risk, atherosclerotic progression, and metabolic-associated fatty liver disease [24–26]. Some researchers have suggested that inflammation may be a critical link between insulin resistance and metabolic disorders such as cardiometabolic multimorbidity and new-onset diabetes [27, 28]. There is limited evidence regarding the combined association of metabolic adiposity indices and systemic inflammation with incident T2DM, especially in middle-aged and older Chinese populations.

To evaluate whether the TyG-ABSI composite index and inflammatory score serve as accessible tools for stratifying type 2 diabetes risk and informing targeted prevention strategies, we used data from the China Health and Retirement Longitudinal Study (CHARLS), a nationally representative prospective cohort (2011–2020), to examine their independent and joint associations with incident T2DM among middle-aged and older Chinese adults.

## Methods

### Study design and population

**Fig. 1:**
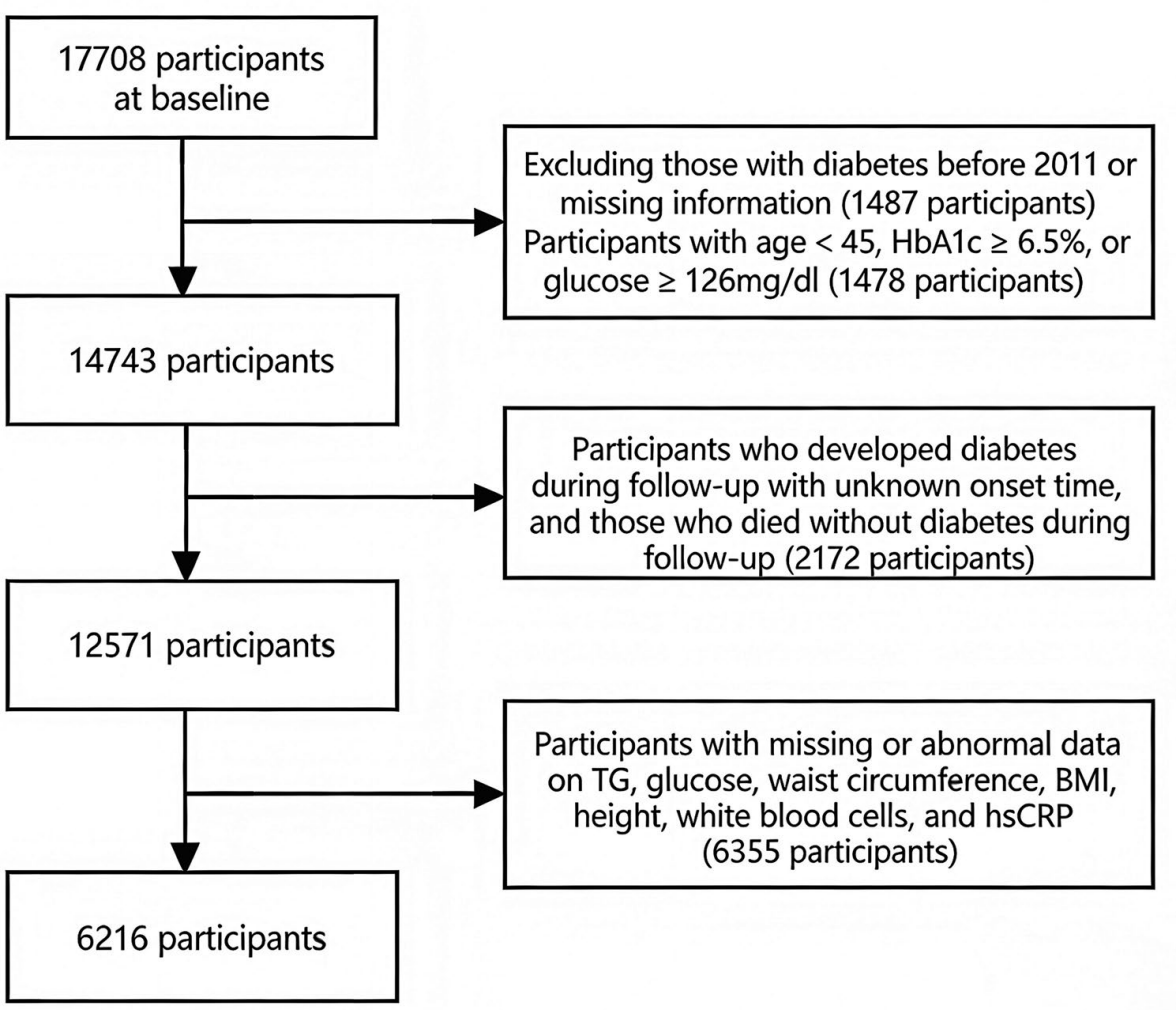
Flow diagram of participant selection. Abbreviations: HbA1c: hemoglobin A1c; TG: triglyceride; BMI: body mass index; hsCRP: high-sensitivity C-reactive protein

This study uses data from the CHARLS. This was a nationwide, representative, longitudinal survey focusing on individuals aged 45 years and older. The baseline survey was conducted between 2011 and 2012, and follow-up assessments were conducted in 2013, 2015, 2018, and 2020. CHARLS employs computer-assisted personal interviewing technology to systematically collect data on respondents’ demographics, health status, lifestyle, socioeconomic conditions, and family support [29]. Following a rigorous participant-screening process, 17,708 participants were enrolled in the baseline survey. Subsequently, 11,492 participants were excluded based on the following criteria: unavailable relevant information or pre-baseline diabetes (*n* = 1,487); age < 45 years, hemoglobin A1c (HbA1c) ≥6.5%, or fasting blood glucose ≥ 126 mg/dL (*n* = 1,478); incident diabetes during follow-up with unknown onset time, or death without diabetes during follow-up (*n* = 2,172); missing or abnormal triglyceride (TG), blood glucose, WC, BMI, height, WBC, or high-sensitivity CRP (hs-CRP) data (*n* = 6,355). Finally, a total of 6,216 participants were included in the final analysis. Figure 1 illustrates a detailed flowchart of the inclusion and exclusion processes. Baseline characteristics between included and excluded participants were compared to assess potential selection bias (Additional file, Table S1). This study was approved by the Institutional Review Board of Peking University (approval no. IRB00001052-11015), and written informed consent was obtained from all participants prior to participation.

### Data collection and measurement

Three tubes of venous blood were collected from each participant by trained staff at the Chinese Center for Disease Control and Prevention after an overnight fast. Among the 6,216 included participants, 415 (6.7%) had non-fasting blood samples at baseline. For these non-fasting subjects, blood samples were still collected and their fasting status recorded; samples were cryopreserved and transported to the Clinical Laboratory of Capital Medical University for centralized testing. TG, total cholesterol (TC), low-density lipoprotein cholesterol (LDL-C), and high-density lipoprotein cholesterol (HDL-C) were measured via enzymatic colorimetric methods; HbA1c via boronate affinity high-performance liquid chromatography; creatinine (Cr) via the rate-blanked and compensated Jaffe method; uric acid (UA) via the UA Plus method; and hs-CRP via immunoturbidimetric assays; leukocyte count via an automated hematology analyzer using the electrical impedance method. All the results were collated and verified by two staff members to ensure accuracy [30]. Height assessment was performed using a calibrated stadiometer, where participants were instructed to stand upright with their heels adjacent and their body weights uniformly distributed. Body weight was measured using a precision-calibrated scale, and all participants were barefoot during the procedure. The BMI was calculated using the following equation: BMI = weight (kg) divided by the height squared (m^2^). The waist circumference was measured at the level of the umbilicus while the participant stood.

### Assessment of TyG-ABSI and inflammatory score

The TyG index was calculated using the formula: TyG = ln[TG (mg/dL) × fasting plasma glucose (FPG, mg/dL)/2] [31]. The TyG-ABSI combines the TyG index with a waist circumference metric standardized by height and BMI [16, 32]. TyG-ABSI was calculated using the following formula: TyG-ABSI = TyG × WC/(BMI^2/3^ × height^1/2^). For each participant, Z-scores were calculated using their biomarker levels (X), study mean (M), and study standard deviation (SD) with the formula: z-score = (X - M)/SD. Subsequently, the inflammatory score was determined by summing the individual Z-scores for hs-CRP and WBC. Prior to this calculation, hs-CRP was logarithmically transformed (logCRP) because of its skewed distribution [26]. Principal component analysis (PCA) was conducted to validate the rationality of equal-weight Z-score summation for the two inflammatory markers (Additional file, Table S2).

### Assessment of diabetes

Diabetes was diagnosed if any of the following criteria were met: fasting blood glucose level ≥ 126 mg/dL (7 mmol/L), random blood glucose level ≥ 200 mg/dL (11.1 mmol/L), HbA1c level ≥ 6.5%, currently using diabetes medication, or self-reported diagnosis of “Have you ever been diagnosed by a doctor with diabetes or high blood sugar?” based on standards published by the American Diabetes Association by 2024 [33]. Despite the publication of updated diagnostic criteria in 2024, the fundamental threshold parameters have remained unchanged since 2010 [34]. Event time was defined as the interval between the baseline assessment date and the date of the first reported occurrence of diabetes; no further follow-up was conducted for participants after they were diagnosed with diabetes. For individuals without any reported diabetes during follow-up, the duration of follow-up was defined as the period from baseline assessment to final follow-up.

### Covariates

All covariate data were systematically collected by certified interviewers using standardized questionnaires, supplemented by objective measurements from structured physical examinations and laboratory assessments. Specifically, the covariates included the following categories: socio-demographic characteristics: age, sex, place of residence (categorized as urban or rural), marital status, and educational level (stratified into four groups: illiteracy, primary school, junior high school, and senior high school and above); lifestyle factors: smoking status (defined as having a history of smoking, including past smoking and current smoking) and drinking status (defined as having a history of drinking, including past drinking and current drinking); disease history: self-reported physician-diagnosed hypertension, heart disease, and stroke; physical examination indicators: resting systolic blood pressure (SBP) and diastolic blood pressure (DBP); laboratory test parameters: blood urea nitrogen, Cr, UA, TC, HDL-C, LDL-C, non-HDL-C, and HbA1c.

### Statistical analysis

Normally distributed measurement data are presented as mean ± standard deviation. One-way ANOVA was performed to compare differences between groups. Pairwise comparisons were carried out with the Bonferroni test for homogeneous variances and the Games-Howell test for heterogeneous variances. For non-normally distributed data, results were presented as medians (Q1, Q3). The Kruskal-Wallis test was used for overall group comparisons, followed by the Dunn-Bonferroni test for pairwise analyses. Count data were reported as absolute values and analyzed using the chi-square test. As an ordinal variable, educational attainment was compared across groups with the Kruskal-Wallis H test, and pairwise comparisons were made using the Bonferroni test.

Multiple imputation by chained equations was performed to address 2.5% of the missing data (155 of 6,216 participants), assuming that the data were missing at random. Detailed summaries of missing data counts and the specific imputation methods employed are provided in Additional file, Table S3.

Potential confounders were screened via univariate Cox proportional hazards regression, with those with *p* < 0.1 retained as candidates (Additional file, Table S4). These candidates were sequentially incorporated into the multivariable Cox model to assess changes in the hazard ratio (HR) of core exposures (TyG-ABSI and Z-score), and variables associated with a > 10% HR change were deemed significant confounders and were retained. Prior to the final model construction, multicollinearity among the retained covariates was assessed using the variance inflation factor (VIF), and no significant multicollinearity was observed (all VIF < 5), supporting the simultaneous inclusion of HDL-C and non-HDL-C.

Multivariate-adjusted Cox proportional hazards models were used to explore the associations of TyG-ABSI, Z-score, and their combination with the incidence of diabetes. TyG-ABSI and Z-scores were binary variables for the single-index analysis and combined categorical variables for the joint analysis. For all analyses, three models were specified: Model 1 without adjustment; Model 2 adjusted for age, sex, smoking status, hypertension, heart disease, and stroke; and Model 3 further adjusted for SBP, UA, HDL, non-HDL-C, and HbA1c. Because TyGABSI is mathematically derived from triglycerides, fasting glucose, waist circumference, BMI, and height, these constituent variables were intentionally excluded from all multivariable models to avoid structural collinearity and overadjustment bias. The diabetes incidence rate per 1,000 person-years was estimated.

Kaplan–Meier curves were used to estimate the cumulative incidence of diabetes, and differences between groups were assessed using the log-rank test. Restricted cubic spline (RCS) functions were used to explore the nonlinear dose-response associations of continuous TyG-ABSI and Z-score with new-onset diabetes after adjusting for the covariates in Model 3. To stabilize the tails of the spline curves, we trimmed the exposures at the 1st and 99th percentiles before fitting the RCS models. The number of knots was optimized based on the lowest Akaike Information Criterion value, with knot locations determined automatically using the data function.

Time-dependent Harrell’s C-index was computed to assess the predictive performance of TyG-ABSI, Z-score, and their combination for diabetes incidence. To evaluate the incremental predictive value of adding TyGABSI and the inflammatory Zscore to the traditional risk factor model, we calculated the continuous Net Reclassification Improvement (NRI) and Integrated Discrimination Improvement (IDI) at the 9 year followup time point. The baseline model consisted of all covariates in Model 3 (age, sex, smoking, hypertension, heart disease, stroke, SBP, UA, HDL, nonHDLC, and HbA1c). The new model additionally included the TyGABSI and Zscore. Confidence intervals for NRI and IDI were estimated using bootstrap with 500 resamples.

Additive and multiplicative interactions were evaluated using three indices: relative excess risk due to interaction (RERI), attributable proportion due to interaction (AP), and synergy index (SI). To explore statistical interdependencies, we conducted exploratory bidirectional mediation analyses with full covariate adjustment. First, we evaluated TyG-ABSI as a mediator between Z-score and incident diabetes using linear regression for the continuous mediator and Cox regression for the outcome. Second, we reciprocally assessed the Z-score as a mediator in the TyG-ABSI-incident diabetes pathway using identical model specifications.

Subgroup analyses were conducted to investigate potential heterogeneity in the association between the TyG-ABSI, Z-score, and their joint effect on diabetes incidence. Sensitivity analyses were conducted to assess the robustness of our findings: (1) reanalyzing TyGABSI and the inflammatory Zscore as tertiles and quartiles; (2) excluding participants who developed incident diabetes within the first 2 years of followup to minimize reverse causality; (3) excluding individuals with nonfasting baseline blood samples; (4) removing HbA1c from the covariate set; (5) additionally adjusting for oral lipidlowering medication use (yes/no); and (6) a comprehensive model that simultaneously excluded HbA1c and adjusted for lipidlowering medication. All models were adjusted for the same covariates as the primary Model 3, except for the specified changes. Finally, considering CHARLS’ multi-stage sampling design, we used survey-weighted Cox regression to verify the associations of TyG-ABSI and inflammatory Z-score with new-onset T2DM (Additional file, Table S11). E-value sensitivity analyses assessed the robustness of significant associations by determining the minimum unmeasured confounding strength necessary to explain the observed effects [35, 36]. All statistical analyses were performed using the R Statistical Software (Version 4.2.2). Two-sided *p*-values < 0.05 indicate statistical significance.

## Results

### Baseline characteristics

The baseline characteristics of the 6,216 participants were analyzed by the joint categories of TyG-ABSI (low/high) and inflammation Z-score (low/high). As shown in Table 1, the high TyG-ABSI/high Z-score subgroup (Group 4) had markedly unfavorable demographic, anthropometric, metabolic, and clinical profiles compared to the low TyG-ABSI/low Z-score subgroup (Group 1). Group 4 participants were significantly older (60.00 ± 8.61 vs. 56.45 ± 8.12 years, *p* < 0.001), more likely to be male (58.3% vs. 52.1%, *p* < 0.001), and had lower educational attainment (illiteracy: 48.3% vs. 43.8%, *p* < 0.001) and higher urban residence (38.3% vs. 31.2%, *p* < 0.001). Anthropometric and cardiovascular parameters showed a clear gradient: Group 4 had higher waist circumference (90.03 ± 9.31 vs. 79.78 ± 8.27 cm), BMI (24.30 ± 3.71 vs. 22.56 ± 3.09 kg/m^2^), SBP (131.86 ± 21.11 vs. 122.87 ± 19.27 mmHg), and DBP (76.64 ± 11.88 vs. 72.62 ± 11.77 mmHg; all *p* < 0.001). The prevalence of hypertension was 2.0-fold higher in Group 4 (30.4% vs. 14.9%), and the prevalence of heart disease was 1.8-fold higher (14.3% vs. 8.1%; both *p* < 0.001). Metabolic and inflammatory markers also differed substantially between the groups. Group 4 had higher TC (203.20 ± 37.65 vs. 184.09 ± 34.17 mg/dL), TG (162.31 vs. 81.95 mg/dL), fasting blood glucose (103.53 ± 10.83 vs. 97.06 ± 10.65 mmol/L), and HbA1c (5.17 ± 0.41% vs. 5.05 ± 0.38%), but lower HDL-C (46.77 ± 13.55 vs. 57.75 ± 14.58 mg/dL; all *p* < 0.001). Inflammatory burden was greater in Group 4: WBC count was 1.4-fold higher (7.08 vs. 5.04 × 10^9^/L) and hs-CRP was elevated (median 1.76 vs. 0.52 mg/L; both *p* < 0.001). Marital status was the only characteristic with no significant between-group differences (*p* = 0.119).

**Table 1:** Baseline characteristics of the study population according to TyG-ABSI and Z-score groups

Variables	Total (n = 6216)	Low TyG-ABSI & Low Z-score (n = 1757)	Low TyG-ABSI & High Z-score (n = 1351)	High TyG-ABSI & Low Z-score (n = 1352)	High TyG-ABSI & High Z-score (n = 1756)	F/χ^2^	P
Age(years)	58.30 ± 8.68	56.45 ± 8.12	57.22 ± 8.57	59.58 ± 8.96^ab^	60.00 ± 8.61^ab^	67.78	<0.001
Gender, n (%)						146.45	<0.001
Male	3452 (55.5)	916 (52.1)	607 (44.9)^a^	905 (66.9)^ab^	1024 (58.3)^abc^		
Female	2764 (44.5)	841 (47.9)	744 (55.1)^a^	447 (33.1)^ab^	732 (41.7)^abc^		
Marital, n (%)						5.85	0.119
Single	651 (10.5)	163 (9.3)	136 (10.1)	147 (10.9)	205 (11.7)		
Married	5565 (89.5)	1594 (90.7)	1215 (89.9)	1205 (89.1)	1551 (88.3)		
Location, n (%)						22.74	<0.001
Urban	2119 (34.1)	549 (31.2)	432 (32)	466 (34.5)	672 (38.3)^ab^		
Rural	4097 (65.9)	1208 (68.8)	919 (68)	886 (65.5)	1084 (61.7)^ab^		
Education, n (%)						45.57	<0.001
Illiteracy	2935 (47.2)	770 (43.8)	594 (44)	722 (53.4)^ab^	849 (48.3)^abc^		
Primary school	1357 (21.8)	389 (22.2)	297 (22)	292 (21.6)^ab^	379 (21.6)^abc^		
Middle school	1263 (20.3)	377 (21.5)	297 (22)	228 (16.9)^ab^	361 (20.6)^abc^		
High school and above	659 (10.6)	220 (12.5)	162 (12)	110 (8.1)^ab^	167 (9.5)^abc^		
Drink, n (%)						49.67	<0.001
no	3724 (59.9)	1001 (57)	733 (54.3)	889 (65.8)^ab^	1101 (62.7)^ab^		
yes	2490 (40.1)	756 (43)	618 (45.7)	462 (34.2)^ab^	654 (37.3)^ab^		
Smoke, n (%)						98.52	<0.001
no	3894 (62.6)	1116 (63.5)	721 (53.4)^a^	970 (71.7)^ab^	1087 (61.9)^bc^		
yes	2322 (37.4)	641 (36.5)	630 (46.6)^a^	382 (28.3)^ab^	669 (38.1)^bc^		
Hypertension, n (%)						122.80	<0.001
no	4785 (77.0)	1487 (84.6)	1057 (78.2)^a^	1024 (75.7)^a^	1217 (69.3)^abc^		
yes	1407 (22.6)	261 (14.9)	288 (21.3)^a^	325 (24)^a^	533 (30.4)^abc^		
Heart disease, n (%)						44.01	<0.001
no	5523 (88.9)	1605 (91.3)	1231 (91.1)	1189 (87.9)^ab^	1498 (85.3)^ab^		
yes	669 (10.8)	143 (8.1)	115 (8.5)	160 (11.8)^ab^	251 (14.3)^ab^		
Stroke, n (%)						11.18	0.021
no	6084 (97.9)	1732 (98.6)	1320 (97.7)	1329 (98.3)	1703 (97)^a^		
yes	118 (1.9)	21 (1.2)	28 (2.1)	22 (1.6)	47 (2.7)^a^		
SBP(mmHg)	127.53 ± 20.63	122.87 ± 19.27	127.23 ± 20.58^a^	128.30 ± 20.51^a^	131.86 ± 21.11^abc^	57.32	<0.001
DBP(mmHg)	74.82 ± 12.07	72.62 ± 11.77	75.18 ± 12.21^a^	74.97 ± 12.09^a^	76.64 ± 11.88^abc^	33.51	<0.001
Height(m)	1.58 ± 0.08	1.59 ± 0.08	1.59 ± 0.08	1.57 ± 0.09^ab^	1.57 ± 0.09^ab^	17.63	<0.001
Weight(kg)	58.29 ± 10.74	56.92 ± 9.71	58.59 ± 10.36^a^	56.99 ± 10.85^b^	60.44 ± 11.55^abc^	40.61	<0.001
WC(cm)	84.58 ± 9.80	79.78 ± 8.27	81.44 ± 9.06^a^	86.86 ± 8.62^ab^	90.03 ± 9.31^abc^	483.83	<0.001
BMI(kg/m^2^)	23.31 ± 3.51	22.56 ± 3.09	23.23 ± 3.53^a^	23.06 ± 3.41^a^	24.30 ± 3.71^abc^	79.10	<0.001
WBC(×10^9^/L)	6.07 ± 1.64	5.04 ± 1.06	7.05 ± 1.49^a^	5.12 ± 1.02^b^	7.08 ± 1.54^ac^	1215.55	<0.001
BUN(mg/dl)	15.58 ± 4.37	15.80 ± 4.45	15.81 ± 4.55	15.30 ± 4.12^ab^	15.40 ± 4.32^ab^	5.51	<0.001
Cr(mg/dl)	0.77 ± 0.18	0.76 ± 0.16	0.79 ± 0.19^a^	0.75 ± 0.17^b^	0.78 ± 0.18^ac^	16.54	<0.001
UA(mg/dl)	4.38 ± 1.19	4.13 ± 1.11	4.49 ± 1.15^a^	4.22 ± 1.15^b^	4.68 ± 1.26^abc^	78.12	<0.001
TC(mg/dl)	192.83 ± 36.53	184.09 ± 34.17	187.69 ± 34.04^a^	195.87 ± 36.72^ab^	203.20 ± 37.65^abc^	97.03	<0.001
TG(mg/dl)	119.32 ± 67.18	81.95 ± 32.49	86.94 ± 35.85^a^	144.42 ± 67.29^ab^	162.31 ± 76.88^abc^	821.54	<0.001
HDL(mg/dl)	52.33 ± 14.89	57.75 ± 14.58	55.19 ± 14.86^a^	49.64 ± 13.80^ab^	46.77 ± 13.55^abc^	209.72	<0.001
LDL(mg/dl)	117.77 ± 33.05	112.52 ± 30.49	116.60 ± 30.91^a^	118.65 ± 34.25^ab^	123.25 ± 35.24^abc^	32.22	<0.001
non-HDL(mg/dl)	140.50 ± 36.02	126.34 ± 32.27	132.50 ± 32.06^a^	146.22 ± 34.74^ab^	156.43 ± 36.12^abc^	269.47	<0.001
HbA1c(%)	5.10 ± 0.39	5.05 ± 0.38	5.09 ± 0.38^a^	5.09 ± 0.39^a^	5.17 ± 0.41^abc^	28.21	<0.001
FBG(mg/dl)	100.03 ± 11.25	97.06 ± 10.65	97.71 ± 11.61	101.64 ± 10.61^ab^	103.53 ± 10.83^abc^	133.51	<0.001
hsCRP(mg/l)	0.90 (0.51, 1.76)	0.52 (0.36, 0.76)	1.59 (0.92, 2.87)^a^	0.59 (0.40, 0.89)^ab^	1.76 (1.06, 2.93)^abc^	2672.00	<0.001
log(hsCRP)	−0.11 (−0.67, 0.57)	−0.65 (−1.02, −0.27)	0.46 (−0.08, 1.05)^a^	−0.53 (−0.92, −0.12)^ab^	0.57 (0.06, 1.08)^abc^	2672.00	<0.001
TyG-ABSI	70.88 ± 6.87	65.30 ± 3.88	65.87 ± 3.76^a^	75.63 ± 4.59^ab^	76.68 ± 4.82^abc^	3218.03	<0.001
WBC-Z score	−0.11 (−0.72, 0.58)	−0.65 (−1.08, −0.23)	0.56 (−0.04, 1.20)^a^	−0.59 (−1.02, −0.17)^ab^	0.50 (−0.04, 1.18)^abc^	2470.00	<0.001
log(hsCRP)-Z score	−0.10 (−0.74, 0.67)	−0.72 (−1.14, −0.29)	0.55 (−0.07, 1.22)^a^	−0.58 (−1.02, −0.11)^ab^	0.67 (0.09, 1.25)^abc^	2672.00	<0.001
Z-score	−0.14 (−1.12, 1.02)	−1.21 (−1.86, −0.67)	0.97 (0.35, 1.74)^a^	−1.00 (−1.61, −0.54)^ab^	1.07 (0.46, 1.92)^ac^	4673.00	<0.001

**Abbreviations**: TyG-ABSI, triglyceride-glucose A Body Shape Index; SBP, systolic blood pressure; DBP, diastolic blood pressure; WC, waist circumference; BMI, body mass index; WBC, white blood cell count; BUN, blood urea nitrogen; Cr, creatinine; UA, uric acid; TC, total cholesterol; TG, triglyceride; HDL, high-density lipoprotein cholesterol; LDL, low-density lipoprotein cholesterol;non-HDL-C,non-high-density lipoprotein cholesterol;HbA1c, glycated hemoglobin; FBG, fasting blood glucose; hsCRP, high-sensitivity C-reactive protein. **Note**: Data are presented as mean ± standard deviation (SD) for normally distributed continuous variables, median (interquartile range, IQR) for non-normally distributed continuous variables, and n (%) for categorical variables. Superscripts a, b, and c indicate significant differences compared with the Low TyG-ABSI & Low Z-score, Low TyG-ABSI & High Z-score, and High TyG-ABSI & Low Z-score group, respectively (*p* < 0.05). Z-score was calculated as the sum of WBC-Z score and log(hsCRP)-Z score. All descriptive statistics are based on the original (preimputation) data

Pairwise post hoc comparisons (see Table 1 footnotes) further revealed that the high TyGABSI/high Zscore subgroup (Group 4) differed significantly from the other three subgroups for most demographic, anthropometric, and metabolic variables (indicated by superscript letters a, b, and c). The baseline characteristics of the participants according to the median values of the TyG-ABSI and Z-scores are presented in Additional file, Tables S5 and S6.

### Association of TyG-ABSI and Z-score with the risk of diabetes

**Fig. 2:**
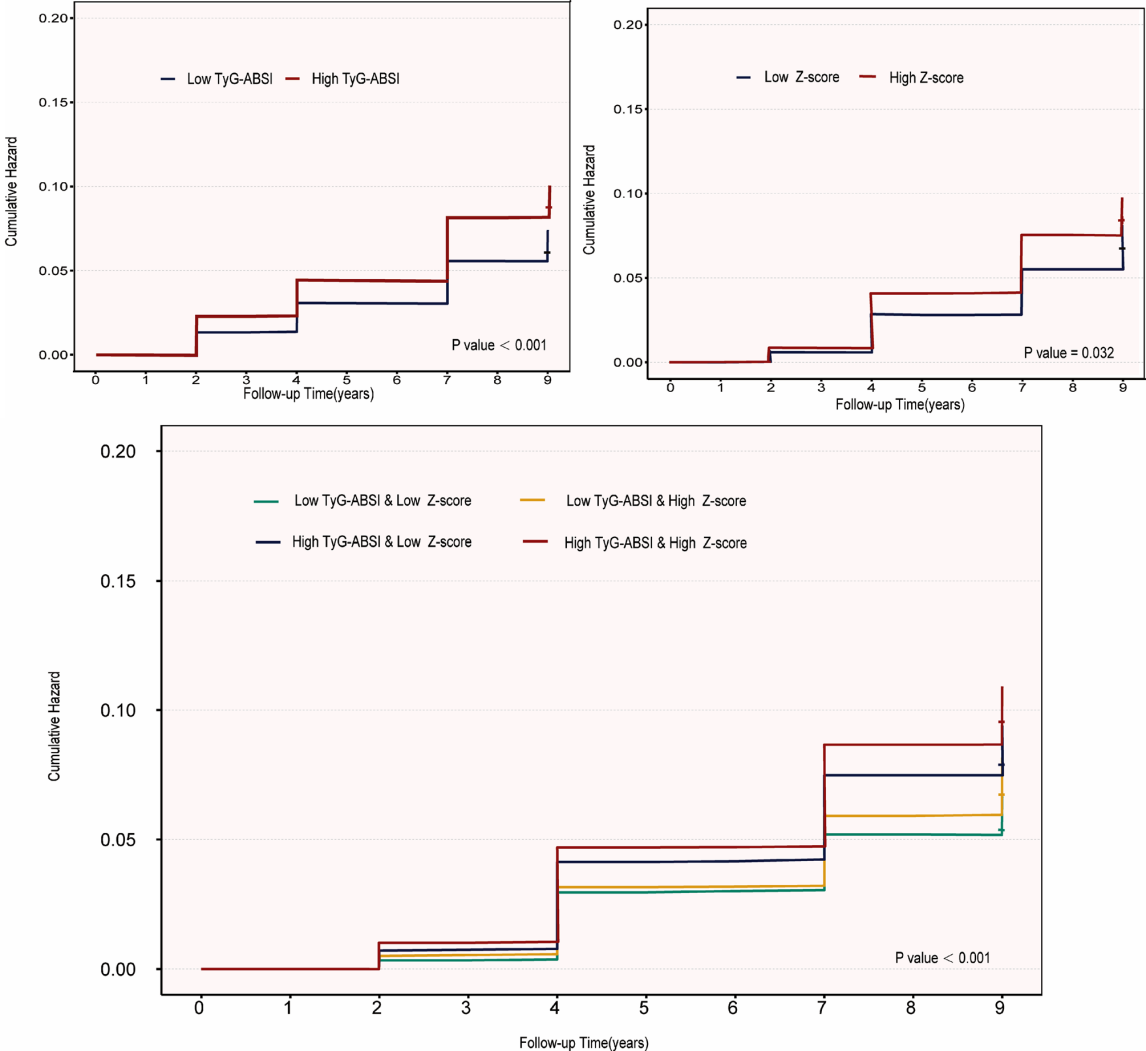
KM plot of incident diabetes risk by TyG-ABSI index and Z-score. Note: the Z-score was calculated as the sum of the white blood cell count Z-score and the log(hscrp) Z-score. Abbreviations: TyG-ABSI, triglyceride-glucose a body shape index

Kaplan–Meier survival curves demonstrated that the cumulative incidence of incident diabetes was progressively elevated in participants with high TyG-ABSI, high Z-score, and concurrently high levels of both TyG-ABSI and Z-score (Fig. 2) (all log-rank *p* < 0.05).

Cox proportional hazards regression analyses with progressive adjustments, presented in Table 2, were used to evaluate the associations between TyG-ABSI, Z-score, and the risk of diabetes. After full adjustment for potential confounders (Model 3), participants with high TyG-ABSI exhibited a 42% increased risk of diabetes (HR: 1.42, 95% confidence interval [CI]: 1.17–1.72; *p* < 0.001) compared to those with low TyG-ABSI. Similarly, participants with high Z-score showed a 21% elevated diabetes risk (HR: 1.21, 95% CI: 1.02–1.44; *p* = 0.032) relative to those with low Z-score. Using the low TyG-ABSI and low Z-score group as the reference, individuals with high TyG-ABSI and low Z-score had a 44% increased diabetes risk (HR: 1.44, 95% CI: 1.09–1.90; *p* = 0.009), while those with concurrent high TyG-ABSI and high Z-score exhibited a 69% higher diabetes risk (HR: 1.69, 95% CI: 1.30–2.21; *p* < 0.001). The low TyG-ABSI and high Z-score group showed a non-significant 23% increased risk (HR: 1.23, 95% CI: 0.93–1.64; *p* = 0.148).

**Table 2:** Risk of incident type 2 diabetes according to TyG-ABSI and Z-score categories

Variable	Event (n)%	**Incidence rate**^**a**^ (95%CI)	**Model 1**^**b**^		**Model 2**^**c**^		**Model 3**^**d**^	
			HR(95 CI%)	P	HR(95 CI%)	P	HR(95 CI%)	P
TyG-ABSI								
Low TyG-ABSI	196 (6.3)	7.19 (6.28~8.15)	Ref		Ref		Ref	
High TyG-ABSI	364 (11.7)	13.71 (12.33~15.20)	1.92 (1.61~2.28)	<0.001	1.77 (1.48~2.12)	<0.001	1.42 (1.17~1.72)	<0.001
Z-score								
Low Z-score	227 (7.3)	8.38 (7.36~9.47)	Ref		Ref		Ref	
High Z-score	333 (10.7)	12.47 (11.17~13.88)	1.49 (1.26~1.77)	<0.001	1.43 (1.21~1.7)	<0.001	1.21 (1.02~1.44)	0.032
TyG-ABSI & Z-score								
Low TyG-ABSI & Low Z-score	95 (5.4)	6.15 (5.04~7.33)	Ref		Ref		Ref	
Low TyG-ABSI & High Z-score	101 (7.5)	8.54 (7.02~10.16)	1.39 (1.05~1.84)	0.021	1.36 (1.03~1.8)	0.033	1.23 (0.93~1.64)	0.148
High TyG-ABSI & Low Z-score	132 (9.8)	11.33 (9.56~13.21)	1.85 (1.42~2.41)	<0.001	1.72 (1.32~2.25)	<0.001	1.44 (1.09~1.9)	0.009
High TyG-ABSI & High Z-score	232 (13.2)	15.58 (13.65~17.66)	2.55 (2.01~3.24)	<0.001	2.31 (1.81~2.95)	<0.001	1.69 (1.3~2.21)	<0.001

**Abbreviations**: TyG-ABSI, triglyceride-glucose A Body Shape Index; HR, hazard ratio; CI, confidence interva. **Note**: Z-score was calculated as the sum of WBC-Z score and log(hsCRP)-Z score^.a^Cases per 1,000 person-years^.b^ Without adjustment;^c^ Adjusted for age, gender, smoking status, hypertension, heart disease and stroke;^d^ Further adjusted for systolic blood pressure (SBP), uric acid (UA), high-density lipoprotein cholesterol (HDL), non-high-density lipoprotein cholesterol (non-HDL-C) and glycated hemoglobin(HbA1c)

### Predictive performance

**Fig. 3:**
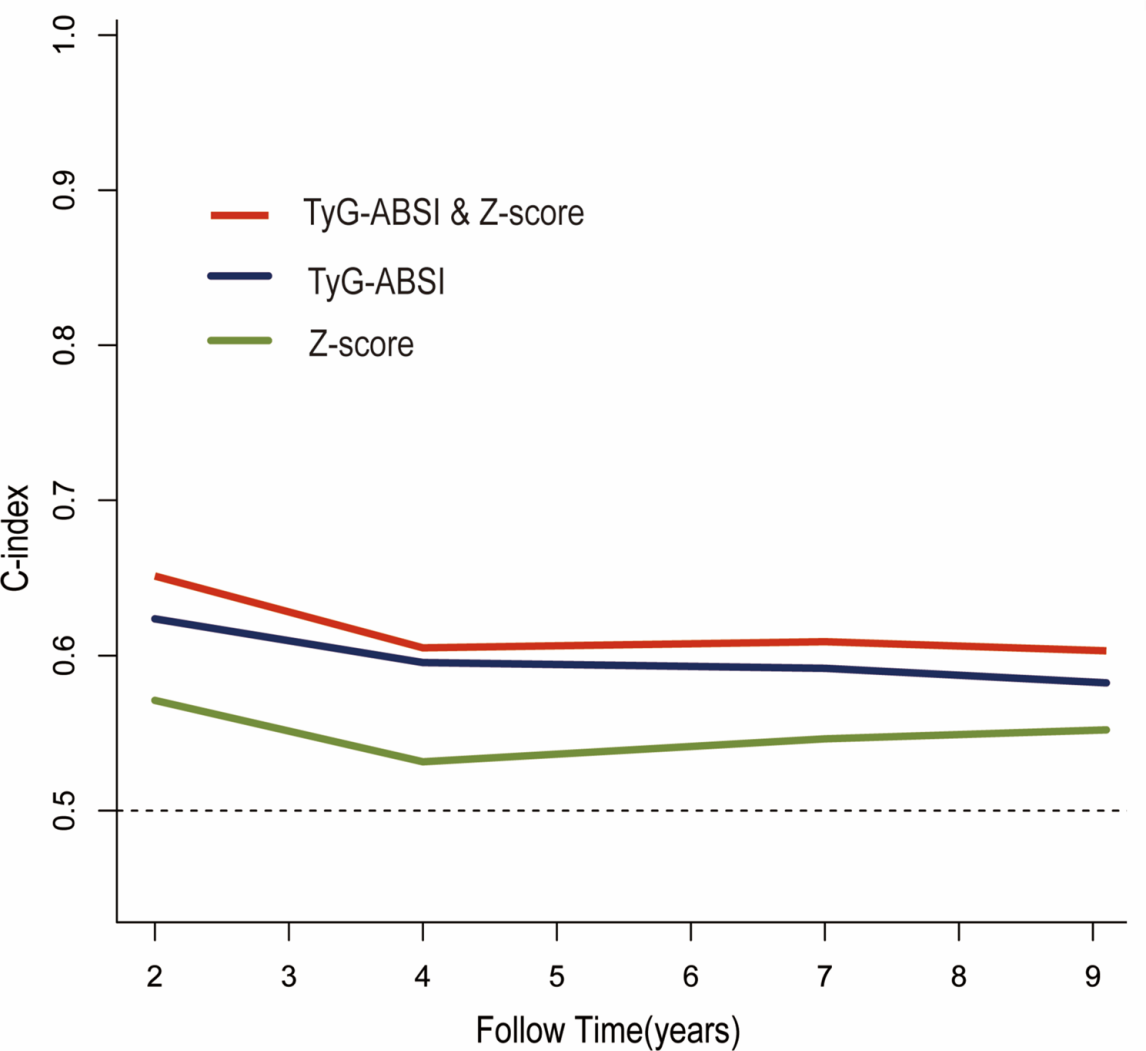
Time-dependent C-index analysis of TyG-ABSI, Z-score, and their combination for diabetes prediction. Abbreviations: TyG-ABSI, triglyceride-glucose a body shape index

Harrell’s C-index was applied to assess time-dependent predictive performance. The combined TyG-ABSI and Z-score yielded C-index values of 0.651 at 2 years, 0.605 at 4 years and 0.610 at 9 years, which were higher than those of TyG-ABSI alone (0.624, 0.596, 0.588) and Z-score alone (0.571, 0.532, 0.556) at all follow-up time points, as shown in Fig. 3. We further calculated the continuous Net Reclassification Improvement (NRI) and Integrated Discrimination Improvement (IDI) at 9 years, comparing each model with the traditional risk factor baseline model (Table 3). The combined TyGABSI and Zscore model yielded a continuous NRI of 0.146 (95% CI: 0.098–0.196, *p* < 0.001) and an IDI of 0.003 (95% CI: 0.001–0.007, *P* =0.004). Adding TyGABSI alone also significantly improved reclassification (NRI = 0.180, 95% CI: 0.100–0.264, *P*  < 0.001) and discrimination (IDI = 0.002, 95% CI: 0.000–0.004, *p* < 0.001). Adding Zscore alone showed smaller but significant improvements (NRI = 0.111, 95% CI: 0.041–0.158, *p* = 0.036; IDI = 0.001, 95% CI: 0.000–0.004, *p* = 0.040).

**Table 3:** Net reclassification improvement (NRI) and integrated discrimination improvement (IDI) for different models compared with the traditional risk factor baseline model (at a 9-year follow-up)

Model	Continuous NRI (95% CI)	P	IDI (95% CI)	P
TyGABSI	0.180 (0.100–0.264)	<0.001	0.002 (0.000–0.004)	<0.001
Zscore	0.111 (0.041–0.158)	0.036	0.001 (0.000–0.004)	0.040
TyGABSI & Zscore	0.146 (0.098–0.196)	<0.001	0.003 (0.001–0.007)	0.004

**Abbreviations:** TyG-ABSI, triglyceride-glucose A Body Shape Index; Z-score was calculated as the sum of WBC-Z score and log(hsCRP)-Z score; HR, hazard ratio; CI, confidence interval. **Note:** The baseline model included age, sex, smoking, hypertension, heart disease, stroke, systolic blood pressure (SBP), uric acid (UA), high-density lipoprotein cholesterol (HDL), non-high-density lipoprotein cholesterol (non-HDL-C) and glycated hemoglobin (HbA1c). NRI and IDI at 9 years were calculated using continuous NRI. Confidence intervals were estimated by bootstrap; *p* values < 0.001 are reported as <0.001, *N* = number of subjects

### Dose-response relationship between TyG-ABSI, Z-score, and incident diabetes risk

**Fig. 4:**
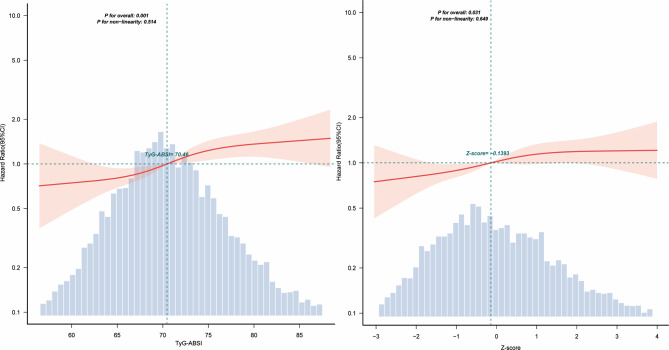
Restricted cubic spline analysis of TyG-ABSI, Z-score, and incident diabetes risk. Note: the Z-score was calculated as the sum of the white blood cell count Z-score and the log(hscrp) Z-score. Abbreviations: TyG-ABSI, triglyceride-glucose a body shape index

RCS functions (with four knots) were incorporated into Cox proportional hazards models to examine the dose–response relationships of continuous TyG-ABSI and the Z-score (Fig. 4). The reference point (HR = 1.0) was set at the median of each continuous exposure. For TyG-ABSI, a stable linear dose–response pattern was similarly observed: the non-linearity test yielded no significant result (*P* for non-linearity = 0.514), and the overall association was statistically significant (*P* for overall = 0.001). The Z-score showed an approximately linear association with incident type 2 diabetes risk; the nonlinearity test was not statistically significant (*P* for nonlinearity = 0.649), while the overall association remained significant (*P* for overall = 0.031). The absence of significant nonlinear relationships for both exposures (all *p* > 0.05) supported their use as continuous linear predictors of diabetes risk without threshold effects.

### Value analysis for robustness of associations with incident diabetes

E-values were calculated to assess the potential impact of unmeasured confounding factors on the associations between TyG-ABSI, Z-score, and their combined levels with incident diabetes using the fully adjusted Model 3 results. The E-values ranged from 1.71–2.77. For high versus low TyG-ABSI, the E-value was 2.19 (exceeding the robustness threshold of 1.82 [35]). For high Z-score versus low Z-score, the E-value was 1.71 (close to the threshold). For the combined high TyG-ABSI and Z-score groups versus the reference group, the E-value was 2.77 (substantially exceeded the threshold). These results indicate that only very strong unmeasured confounders could nullify the associations of TyG-ABSI and combined exposure to diabetes risk, while the Z-score association requires moderate unmeasured confounding to be attenuated, overall supporting the credibility of our findings.

### Subgroup analyses

**Fig. 5:**
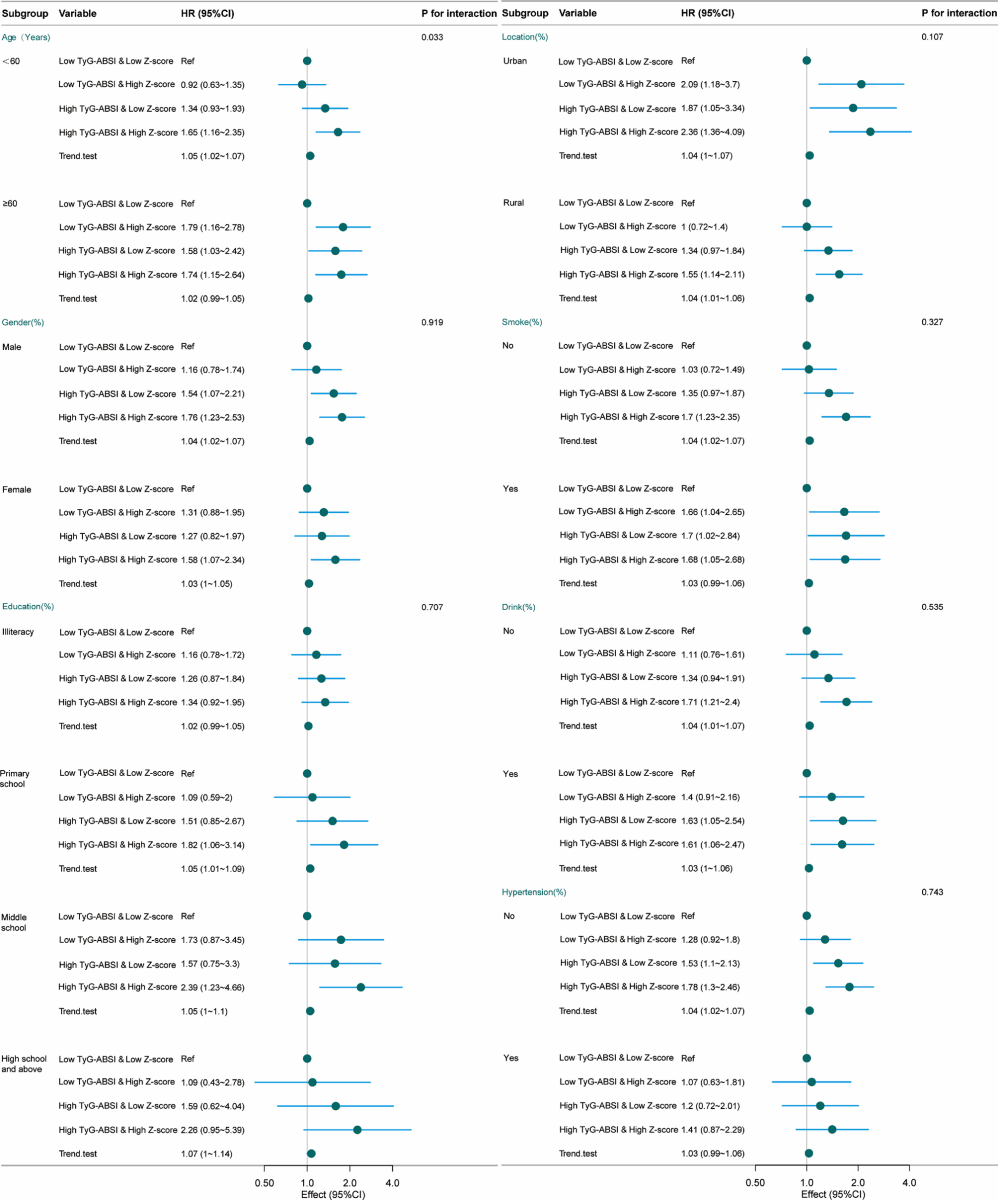
Subgroup analysis of the association between combined TyG-ABSI and Z-score levels and incident diabetes risk. Note: the Z-score was calculated as the sum of the white blood cell count Z-score and the log(hscrp) Z-score. Abbreviations: TyG-ABSI, triglyceride-glucose a body shape index

Stratified analyses were performed to assess the association of TyG-ABSI and Z-scores with diabetes events across various subgroups. The associations between TyG-ABSI and Z-scores and the risk of diabetes in most subgroups were consistent with the main results. A significant interaction was observed with age (*P* for interaction = 0.033), whereas no significant interactions were detected in the other subgroups (Fig. 5). Similar results were obtained using TyG-ABSI (Additional file, Fig S1), with no significant subgroup interactions (all *p* > 0.05). The association of Z-scores (Additional file, Fig S2), with diabetes risk aligned well with the main findings, and no significant subgroup interactions were noted (all *p* > 0.05).

### Sensitivity analyses

Multiple sensitivity analyses were conducted to test the robustness of the associations between TyGABSI, the inflammatory Zscore, and incident diabetes. First, reanalysing TyGABSI as tertiles and quartiles yielded results consistent with the continuous analysis, confirming that higher TyGABSI levels were independently associated with an increased risk of diabetes (Additional file, Table S7). Second, excluding participants who developed diabetes within the first two years of followup to minimise reverse causality did not change the significant associations (Additional file, Table S8). Third, the findings remained robust after removing individuals with nonfasting baseline blood samples (Additional file, Table S9). Fourth, when HbA1c was excluded from the covariate set to avoid potential confounding by baseline glycaemic status, the predictive effects of TyGABSI and the Zscore remained statistically significant (Additional file, Table S10). Fifth, additional adjustment for oral lipidlowering medication use did not materially alter the combined predictive value of TyGABSI and the Zscore (Additional file, Table S10). Sixth, a comprehensive sensitivity model that simultaneously excluded HbA1c and adjusted for lipidlowering medication further confirmed the robustness of the primary findings (Additional file, Table S10). Seventh, survey-weighted Cox regression accounting for CHARLS’ sampling design yielded results consistent with the primary analysis. The hazard ratio of the high TyG-ABSI and high Z-score group (vs. low-low reference) was 1.90 (95% CI: 1.38–2.61, *p* < 0.001). Results for all joint exposure groups are listed in Additional file Table S11.

### Assessment of additive and multiplicative interactions between TyG-ABSI and Z-score

Following comprehensive adjustment for potential confounders, we observed that the 95% CI for the multiplicative scale included 1, while those for RERI and AP included 0, and the interval for SI also included 1. These results indicate the absence of statistically significant additive or multiplicative interactions between TyG-ABSI and the Z-score for incident diabetes (Additional file, Table S12).

### Exploratory mediation analysis

We performed exploratory bidirectional mediation analyses to examine statistical interdependencies between TyGABSI and the inflammatory Zscore. As both indicators were measured concurrently at baseline, causal inference could not be drawn. Detailed results, including mediation proportions of approximately 5–6%, are presented in the Supplementary Materials (Additional file, Fig S3).

## Discussion

This study used data from the 2011 to 2020 CHARLS, a large nationally representative prospective cohort of middle-aged and older Chinese adults [29], to systematically examine the independent and joint associations of the TyG-ABSI composite index and inflammatory score (Z-score) with incident T2DM. The key findings of this study are as follows: first, both high TyG-ABSI and high Z-score were independently associated with an increased risk of T2DM after full adjustment for potential confounders; second, the joint effect of high TyG-ABSI and high Z-score exerted a more pronounced impact on T2DM risk compared to either factor alone, with a 69% higher risk observed relative to the reference group (low TyG-ABSI and low Z-score); third, dose-response analyses using RCS revealed approximately linear relationships between continuous TyG-ABSI, Z-score, and T2DM risk, without significant threshold effects; fourth, the combined index demonstrated superior discriminative ability (Harrell’s C-index) and significantly improved risk reclassification (NRI) and overall discrimination (IDI) compared with traditional risk factors alone; finally, E-value analyses and sensitivity analyses confirmed the robustness of these associations, while subgroup analyses identified age as a significant effect modifier.

As a validated insulin resistance surrogate, the TyG index is closely linked to T2DM [10], and its combination with anthropometric indicators enhances metabolic disease prediction. Consistent with this rationale, a Chinese cohort study by Huang et al. using CHARLS data demonstrated that TyG-BMI (another TyG-derived index) was independently associated with new-onset diabetes, confirming the reliability of TyG-based composite indices for diabetes risk assessment [37]. The ABSI overcomes the limitations of traditional anthropometric measures by standardizing waist circumference, which more accurately reflects abdominal fat distribution, independent of total body size. Recent evidence further supports the clinical utility of ABSI in reflecting visceral adiposityrelated health outcomes [38]. Although one study reported that ABSI was significantly associated with incident T2DM in middle-aged and elderly Chinese individuals, its predictive efficacy was relatively low, as indicated by receiver operating characteristic curve analysis [39].

Similar to TyG-BMI, TyG-ABSI integrates the TyG index with ABSI, thereby comprehensively capturing metabolic dysfunction and adiposity, which are two key pathological determinants of T2DM. Previous studies have shown that TyG-ABSI outperforms traditional TyG-derived indices and single anthropometric or metabolic markers in predicting cardiovascular disease, mortality, and stroke [16, 18]. Our study complements this evidence by confirming that the TyG-ABSI is a valuable predictor of increased T2DM risk after full adjustment.

Consistent with our hypothesis, the inflammatory score (Z-score, combining log-transformed hs-CRP and WBC Z-scores) was independently associated with elevated T2DM risk, with a high Z-score corresponding to a 21% higher risk. This is consistent with a large body of evidence highlighting the critical role of inflammation in T2DM development [40, 41]. Inflammation has been recognized as a key link between insulin resistance and metabolic disorders, with elevated levels of inflammatory markers such as CRP and WBC count contributing to pancreatic β-cell dysfunction, impaired insulin signaling, and accelerated progression to T2DM [21, 42]. Our findings support the utility of a composite inflammatory score for assessing systemic inflammation and predicting T2DM risk, as it integrates multiple inflammatory markers to capture the cumulative burden of inflammation, rather than relying on a single indicator.

Although additive and multiplicative interaction analyses failed to yield statistically significant results, a descriptive joint association analysis revealed that the coexistence of high TyG-ABSI and inflammatory Z-scores was linked to the highest T2DM risk. This finding indicates a potential combined association between metabolic adiposity dysfunction and systemic inflammation, which warrants further validation in larger-scale cohorts and is consistent with accumulating evidence that metabolic abnormalities and chronic low-grade inflammation are inherently interconnected and mutually reinforcing in T2DM pathogenesis [43, 44].

As a composite index capturing insulin resistance and central obesity, which are core metabolic-adiposity disorders, TyG-ABSI is closely associated with inflammatory pathway activation in metabolic tissues [44]. Specifically, excess adiposity promotes the recruitment of pro-inflammatory macrophages and the secretion of cytokines such as tumor necrosis factor (TNF)-α, interleukin(IL)-1β, and IL-6, which impair insulin signaling, exacerbate insulin resistance, and perpetuate metabolic disturbances. This forms a vicious cycle that accelerates T2DM progression [43], and our study extends this understanding by highlighting that the joint effect of TyG-ABSI and the inflammatory score can better characterize T2DM risk, underscoring the joint contribution between metabolic adiposity dysfunction and systemic inflammation in disease development.

Notably, our exploratory bidirectional mediation analyses suggested modest statistical interdependencies (approximately 56%). The relatively modest mediation effects may stem from the multifaceted, multiorgan mechanisms underlying the crosstalk between obesity, inflammation, and T2DM, beyond the scope of our inflammatory Z-score and metabolic adiposity index. Existing evidence indicates that metabolic dysfunction and chronic tissue inflammation interact via diverse pathways including adipose tissue hypoxia, macrophage-derived exosomal miRNAs, gut microbiota dysbiosis, and hepatic steatosis [45, 46]. For instance, macrophage-derived exosomes can directly modulate insulin sensitivity in vivo and in vitro [46], while chronic tissue inflammation drives metabolic derangements through transcriptional regulation and signaling pathway crosstalk [45]. Given the complexity of these biological pathways, non-significant additive and multiplicative interactions are plausible. Binary interaction models may not fully capture the intricate relationship between metabolic adiposity dysfunction and inflammation, because their combined effects likely involve nonlinear regulatory networks and context-dependent mechanisms that require more sophisticated analytical frameworks for dissection.

We further explored the dose-response relationships of TyG-ABSI and inflammatory Z-score with T2DM risk using RCS analysis. Our results revealed a continuous, linear dose-response association for both indices across their entire distribution (all *P* for nonlinearity > 0.05). Specifically, T2DM risk rose incrementally with increasing values of TyG-ABSI and inflammatory Z-score, with no evidence of critical inflection points or threshold effects throughout the exposure range. Notably, this finding differs from the nonlinear patterns reported for other metabolic markers and metabolic-inflammatory pathways. At the population level for metabolic markers, Cai et al. [47] demonstrated a distinctive nonlinear association between Metabolic Score for Insulin Resistance (METS-IR) and T2DM among normal-weight middle-aged and older adults, with an inflection point at 37.24, which was positively associated with T2DM risk below this value (HR: 1.18, 95% CI: 1.14–1.21) but showed no significant association. For metabolic-inflammatory pathways at the mechanistic level, threshold-dependent dynamics are well recognized in a contrasting manner; adipose tissue inflammation accelerates insulin resistance only after fat accumulation, and proinflammatory mediator secretion reaches critical biological levels. Lee & Olefsky [45] clarified that this mechanistic threshold effect arises from adipocyte hypoxia (intracellular oxygen tension < 1.4% stabilizes HIF-1α, initiating a cascade of chemokine secretion and monocyte recruitment that amplifies inflammation and insulin resistance), while Zatterale et al. [48] reinforced that macrophage infiltration beyond critical levels acts as a key trigger linking obesity to insulin resistance.

In contrast, T2DM risk in our cohort increased linearly with TyG-ABSI. The METS-IR referenced in the study by Cai et al. [47] includes BMI, a general measure of overall fat mass that fails to distinguish visceral fat from subcutaneous fat, whereas TyG-ABSI integrates ABSI, an index specifically optimized to reflect visceral fat distribution [13]. Visceral fat hypertrophy induces adipose tissue hypoxia, macrophage infiltration, and proinflammatory cytokine secretion (e.g., IL-6, TNF-α) [44, 49], which are hallmarks of the chronic low-grade inflammation prevalent in middle-aged and older adults [50]. This persistent inflammatory state disrupts metabolic homeostasis and exacerbates insulin resistance, while insulin resistance further promotes metabolic derangements that sustain inflammatory activation—creating a self-perpetuating cycle that drives continuous risk increments. Moreover, the study population in Cai et al. [47] consisted of normal-weight middle-aged and older adults, which may also contribute to the inconsistent findings in RCS analyses. Further dedicated comparative studies are needed to validate TyG-ABSI’s superior sensitivity to visceral fat–related metabolic dysregulation in aging populations, compared with METS-IR and other conventional indices. Additionally, as clinical composite indices, TyG-ABSI and the inflammatory Z-score capture integrated population-level risk, distinct from the threshold-dependent dynamics of individual molecular/cellular processes [45, 48]. These findings have significant clinical implications. Unlike METS-IR or metabolic-inflammatory pathways, any increase in TyG-ABSI or inflammatory Z-scores contributed to T2DM risk in our middle-aged and older cohorts. This underscores the need for early, continuous intervention across the entire range of these markers, and timely risk modification in this population can significantly reduce T2DM incidence.

The time-dependent Harrell’s C-index for the combined index was highest at 2 years (0.651) and gradually declined to approximately 0.59–0.61 at later follow-up points. This pattern is expected, as long-term risk prediction for a multifactorial disease such as T2DM becomes inherently more challenging. Nevertheless, the combined index consistently outperformed either single index across all time points. The continuous NRI of 0.146 (95% CI: 0.098–0.196, *p* < 0.001) indicated a moderate but statistically significant improvement in risk reclassification over traditional risk factors alone. Although the NRI for the combined index was slightly lower than that for TyG-ABSI alone (0.180), this is likely due to the sensitivity of NRI to the choice of risk thresholds; the combined index showed the highest IDI (0.003, *p* = 0.004), reflecting better overall discrimination. From a clinical perspective, although the TyG-ABSI formula requires calculation, the combined index may still be useful for risk stratification. In resource-limited primary care, simpler measures such as BMI remain practical; however, where feasible, the combined index could offer additional information for early detection of high-risk individuals. The observed discriminative ability and reclassification improvement support its potential application in targeted prevention strategies.

Subgroup analyses identified age as a significant effect modifier of the associations between TyG-ABSI, inflammatory Z-score, and incident T2DM risk, with no significant interactions noted for other subgroups (e.g., sex, residential area, lifestyle factors). This age-specific modification is mechanistically consistent with the findings of Qiu et al. [51], despite superficial discrepancies. MRI-based quantification of visceral and subcutaneous fat areas revealed a weakened association between central obesity and hyperglycemia with advancing age (odds ratio [OR] = 5.220 for 40–49 years; OR = 1.596 for 60–65 years). The strengthened association of the four subgroups in older adults was derived from the composite advantage of the integrated indices. Incorporating visceral fat distribution (ABSI), insulin resistance (TyG index), and inflammatory status (Z-score), this approach aligns with older adults’ pathological features (β-cell dysfunction, persistent insulin resistance, aberrant fat distribution, as confirmed by Qiu et al. [51]), accurately capturing risk increments under chronic metabolic stress and overcoming the predictive limitations of single obesity-related biomarkers. In comparison, Tian et al. [52] showed that most single metabolic factors had age-attenuated associations with T2DM. In contrast, our integrated TyG-ABSI and inflammatory Z-score indices exhibited a robust association in older adults, overcoming the diluted predictive efficacy of single biomarkers owing to the coexistence of multiple risk factors. This confirms that the combined indices are better suited for capturing the complex risk pathways in older adults and offer a more precise tool for risk stratification.

The robustness of our findings was supported by several key methodological strengths. First, it was based on a nationally representative CHARLS cohort, which enhances the generalizability of our results to middle-aged and older Chinese adults. Second, we applied rigorous eligibility criteria and comprehensively adjusted for a broad spectrum of potential confounders, thereby minimizing the confounding bias. Third, multiple imputation were used to manage missing data, preserve the statistical power, and reduce related biases. Fourth, E-value analyses suggested that substantial unmeasured confounding factors would be required to reverse the observed associations, reinforcing the credibility of our results. Finally, extensive sensitivity analyses (including tertile/quartile categorizations, exclusion of earlyonset diabetes or nonfasting samples, removal of HbA1c, and adjustment for lipidlowering medication) consistently confirmed the stability of our main findings.

Despite these strengths, this study had some limitations. First, our inflammatory score was constructed using only hs-CRP and WBC counts; other critical inflammatory markers were unavailable, which may have underestimated the overall inflammatory burden. Second, we did not examine the potential interactions between the components of the TyG-ABSI or the inflammatory score, thus limiting further mechanistic interpretations. Third, the CHARLS cohort included only Chinese adults aged 45 years and older, restricting its generalizability to younger or other ethnic populations. Fourth, the long-term predictive value beyond 9 years of follow-up remains unclear and requires extended follow-up in future research. Fifth, although we compared baseline characteristics between included and excluded participants and adjusted for all measured confounders, residual selection bias due to unmeasured factors cannot be completely ruled out. Nevertheless, any remaining bias is likely to be conservative. Sixth, as this was an observational study, causal relationships cannot be established, and future randomized controlled trials are warranted to verify causality and clinical applicability.

## Conclusion

This study demonstrated that TyG-ABSI and inflammatory scores were independently associated with T2DM in middle-aged and older Chinese adults, and their combined exposure conferred a significantly elevated risk. Linear dose–response and mediation analyses suggest statistical associations between metabolic adiposity dysfunction and systemic inflammation in relation to T2DM, and TyG-ABSI and inflammatory scores represent simple, cost-effective biomarkers for risk stratification and targeted preventive strategies. Routine application in primary care may facilitate early identification of high-risk individuals and enable timely interventions. Further multicenter prospective studies are warranted to validate these findings and elucidate the underlying biological mechanisms.

## Electronic supplementary material

Below is the link to the electronic supplementary material.

Supplementary Material 1

## Data Availability

The data supporting the findings of this study are available from the China Health and Retirement Longitudinal Study (CHARLS) Repository. Data access can be obtained by registering and submitting a request via the official CHARLS website at http://charls.pku.edu.cn.

## Abbreviations

ABSI: A Body Shape Index
AP: Attributable proportion due to interaction
BMI: Body mass index
CHARLS: China Health and Retirement Longitudinal Study
Cr: Creatinine
CRP: C-reactive protein
DBP: Diastolic blood pressure
HDL-C: High-density lipoprotein cholesterol
HR: Hazard ratio
hs-CRP: High-sensitivity C-reactive protein
LDL-C: Low-density lipoprotein cholesterol
METS-IR: Metabolic Score for Insulin Resistance
OR: Odds ratio
RCS: Restricted cubic spline
RERI: Relative excess risk due to interaction
SBP: Systolic blood pressure
SI: Synergy index
T2DM: Type 2 diabetes mellitus
TC: Total cholesterol
TG: Triglyceride
TyG-ABSI: Triglyceride-glucose A Body Shape Index
UA: Uric acid
VIF: Variance inflation factor
WBC: White blood cell count
WC: Waist circumference
