# Supplementary material for: Joint association of TyG-ABSI composite index and inflammatory score with diabetes in middle-aged and older Chinese adults: a prospective cohort study from CHARLS (2011–2020)
Source: BMC Endocr Disord. 2026 Jun 19;26:209. doi: 10.1186/s12902-026-02370-3 (PMC13374195; doi:10.1186/s12902-026-02370-3)
Supplement: Supplementary file 1 — Supplementary Material 1 [file 12902_2026_2370_MOESM1_ESM.pdf]

Table S1 Comparison of baseline characteristics between included participants (n=6,216) and excluded participants without prevalent diabetes (n=9,149)

**Variables**	**Total (n = 15365)**	**Excluded (n = 9149)**	**Included (n = 6216)**	***P***	**Valid(n)**	**Missing(%)**
Age(years)	58.60 ± 10.22	58.81 ± 11.15	58.30 ± 8.68	0.002	9042	1.17
Gender, n (%)				< 0.001	9147	0.02
Male	7992 (52.0)	4540 (49.6)	3452 (55.5)			
Female	7371 (48.0)	4607 (50.4)	2764 (44.5)			
Marital, n (%)				< 0.001	9117	0.35
Single	1963 (12.8)	1312 (14.4)	651 (10.5)			
Married	13370 (87.2)	7805 (85.6)	5565 (89.5)			
Location, n (%)				< 0.001	9149	0.00
Urban	6112 (39.8)	3993 (43.6)	2119 (34.1)			
Rural	9253 (60.2)	5156 (56.4)	4097 (65.9)			
Education, n (%)				< 0.001	9097	0.57
Illiteracy	6936 (45.3)	4001 (44)	2935 (47.2)			
Primary school	3233 (21.1)	1876 (20.6)	1357 (21.8)			
Middle school	3195 (20.9)	1932 (21.2)	1263 (20.3)			
High school and above	1947 (12.7)	1288 (14.2)	659 (10.6)			
Drink, n (%)				0.079	9149	0.00
no	9077 (59.1)	5353 (58.5)	3724 (59.9)			
yes	6286 (40.9)	3796 (41.5)	2490 (40.1)			

Smoke, n (%)				< 0.001	9149	0.00
no	9329 (60.7)	5435 (59.4)	3894 (62.6)			
yes	6036 (39.3)	3714 (40.6)	2322 (37.4)			
Hypertension, n (%)				0.137	8955	2.12
no	11612 (76.7)	6827 (76.2)	4785 (77.3)			
yes	3535 (23.3)	2128 (23.8)	1407 (22.7)			
Heart disease, n (%)				0.454	8953	2.14
no	13474 (89.0)	7951 (88.8)	5523 (89.2)			
yes	1671 (11.0)	1002 (11.2)	669 (10.8)			
Stroke, n (%)				< 0.001	8993	1.71
no	14829 (97.6)	8745 (97.2)	6084 (98.1)			
yes	366 (2.4)	248 (2.8)	118 (1.9)			
SBP(mmHg)	128.82 ± 21.47	130.21 ± 22.27	127.53 ± 20.63	< 0.001	5673	37.99
DBP(mmHg)	75.25 ± 12.31	75.71 ± 12.56	74.82 ± 12.07	< 0.001	5673	37.99
Height(m)	1.58 ± 0.09	1.58 ± 0.10	1.58 ± 0.08	0.638	5589	38.91
Weight(kg)	58.30 ± 11.62	58.30 ± 12.52	58.29 ± 10.74	0.964	5622	38.55
WC(cm)	83.64 ± 12.42	82.62 ± 14.71	84.58 ± 9.80	< 0.001	5648	38.27
BMI(kg/m^2)	22.90 (20.64, 25.48)	22.78 (20.47, 25.43)	23.01 (20.80, 25.53)	0.001	5538	39.47
WBC(×10^9/L)	6.20 ± 2.13	6.44 ± 2.82	6.07 ± 1.64	< 0.001	3360	63.27
BUN(mg/dl)	15.69 ± 4.63	15.88 ± 5.07	15.58 ± 4.37	0.003	3448	62.31

Cr(mg/dl)	0.78 ± 0.24	0.80 ± 0.32	0.77 ± 0.18	< 0.001	3423	62.59
UA(mg/dl)	4.44 ± 1.24	4.54 ± 1.33	4.38 ± 1.19	< 0.001	3447	62.32
TC(mg/dl)	191.48 ± 37.38	189.04 ± 38.74	192.83 ± 36.53	< 0.001	3439	62.41
TG(mg/dl)	122.02 ± 75.95	126.89 ± 89.45	119.32 ± 67.18	< 0.001	3440	62.40
HDL(mg/dl)	51.86 ± 15.03	51.02 ± 15.25	52.33 ± 14.89	< 0.001	3446	62.33
LDL(mg/dl)	116.39 ± 33.80	113.90 ± 34.98	117.77 ± 33.05	< 0.001	3445	62.35
non-HDL(mg/dl)	139.62 ± 37.03	138.02 ± 38.73	140.50 ± 36.02	0.002	3439	62.41
HbA1c(%)	5.09 ± 0.40	5.07 ± 0.42	5.10 ± 0.39	0.001	3551	61.19
FBG(mg/dl)	99.91 ± 12.10	99.69 ± 13.51	100.03 ± 11.25	0.192	3421	62.61
hsCRP(mg/l)	0.97 (0.53, 2.06)	1.15 (0.57, 3.01)	0.90 (0.51, 1.76)	< 0.001	3447	62.32

Abbreviations: SBP, systolic blood pressure; DBP, diastolic blood pressure; WC, waist circumference; BMI, body mass index; WBC, white blood cell count; BUN, blood urea nitrogen; Cr, creatinine; UA, uric acid; TC, total cholesterol; TG, triglyceride; HDL, high-density lipoprotein cholesterol; LDL, low-density lipoprotein cholesterol; non-HDL-C, non-high-density lipoprotein cholesterol; HbA1c, glycated hemoglobin; FBG, fasting blood glucose; hsCRP, high-sensitivity C-reactive protein. **Note:** "Valid" (n) indicates the number of excluded participants with nonmissing data for each variable. Missing (%) is calculated as (9,149 – valid n) / 9,149 × 100. For variables with high missing rates (>60%), comparisons should be interpreted with caution, as they are based on a subset of the excluded group.

Table S2 Principal component analysis (PCA) of the two inflammatory markers (log-transformed hs-CRP and white blood cell count) to validate the equal-weight Z-score approach

**Measure**	**Value**
Kaiser- Meyer- Olkin (KMO) measure of sampling adequacy	0.500
Bartlett's test of sphericity (χ^2, ***df, P***)	252.13, ***df***=1, ***P***<0.001
Eigenvalue of first principal component (PC1)	1.199

**Measure**	**Value**
Variance explained by PC1 (%)	59.97%
Factor loadings (log(hsCRP), WBC)	0.774, 0.774
Component score coefficients (log(hsCRP), WBC)	0.646, 0.646

Abbreviations: hsCRP, high-sensitivity C-reactive protein; WBC, white blood cell count. **Note:** PCA was performed on the standardized z-scores of log-transformed hs-CRP and WBC. Both markers had identical loadings and coefficients on the first principal component, supporting the use of an equal-weight Z-sum score as the inflammatory score.

Table S3 The proportion of missing covariates and imputation methods

**Variable**	**Number of missing data**	**Percentage of missing data (%)**	**Imputation methods**
Age	11	0.18	norm
Education	2	0.03	mlogit
Drink	2	0.03	logreg
Hypertension	24	0.39	logreg
Heart disease	24	0.39	logreg
Stroke	14	0.23	logreg
SBP	44	0.71	norm
DBP	45	0.72	norm
HbA1c	46	0.74	norm

Abbreviations: SBP,systolic blood pressure;DBP,diastolic blood pressure;HbA1c,hemoglobin A1c.

Table S4 Univariate Cox Proportional Hazards Regression Analyses of Associations Between Potential Confounders, Core Exposures (TyG-ABSI, Z-score), and Incident Type 2 Diabetes

**Item**	**HR(95%CI)**	***P***
Age(years)	1.01 (1.00,1.02)	0.023
Gender:Female vs Male	0.87 (0.73,1.03)	0.093
Marita: Married vs Single	0.87 (0.67,1.12)	0.282
Education		0.128
Illiteracy	Ref	
Primary school	0.91 (0.74,1.13)	
Middle school	0.78 (0.62,0.98)	
High school and above	0.81 (0.61,1.09)	

Location:Rural vs Urban	1.14 (0.95,1.36)	0.155
Drink: yes vs no	0.9 (0.76,1.06)	0.207
Smoke: yes vs no	0.82 (0.69,0.97)	0.023
Hypertension: yes vs no	1.92 (1.61,2.28)	< 0.001
Heart disease: yes vs no	1.73 (1.39,2.16)	< 0.001
Stroke: yes vs no	1.67 (1.03,2.71)	0.053
SBP(mmHg)	1.0081 (1.0044,1.0119)	< 0.001
DBP(mmHg)	1.01 (1.01,1.02)	< 0.001
BUN(mg/dl)	1.0046 (0.986,1.0237)	0.629
Cr(mg/dl)	1.14 (0.72,1.82)	0.572
UA(mg/dl)	1.07 (1,1.15)	0.047
TC(mg/dl)	1.0046 (1.0024,1.0068)	< 0.001
HDL(mg/dl)	0.98 (0.98,0.99)	< 0.001
LDL(mg/dl)	1.0047 (1.0023,1.0071)	< 0.001
non-HDL(mg/dl)	1.0071 (1.005,1.0092)	< 0.001
HbA1c(%)	2.83 (2.29,3.48)	< 0.001
TyG-ABSI	1.05 (1.04,1.06)	< 0.001
Z-score	1.17 (1.11,1.23)	< 0.001

Abbreviations: TyG-ABSI, triglyceride- glucose A Body Shape Index; SBP, systolic blood pressure; DBP, diastolic blood pressure; BUN, blood urea nitrogen; Cr, creatinine; UA, uric acid; TC, total cholesterol; HDL, high-density lipoprotein cholesterol; LDL, low-density lipoprotein cholesterol;non-HDL-C,non-high-density lipoprotein cholesterol;HbA1c, glycated hemoglobin.
Note: Z-score was calculated as the sum of WBC-Z score and log(hsCRP)-Z score.

Table S5 Baseline characteristics of participants stratified by low and high TyG-ABSI levels

**Variables**	**Total (n = 6216)**	**Low TyG-ABSI (n = 3108)**	**High TyG-ABSI (n = 3108)**	***P***
Age(years)	58.30 ± 8.68	56.79 ± 8.32	59.82 ± 8.77	< 0.001
Gender, n (%)				< 0.001
Male	3452 (55.5)	1523 (49)	1929 (62.1)	
Female	2764 (44.5)	1585 (51)	1179 (37.9)	
Marital, n (%)				0.028
Single	651 (10.5)	299 (9.6)	352 (11.3)	
Married	5565 (89.5)	2809 (90.4)	2756 (88.7)	
Location, n (%)				< 0.001
Urban	2119 (34.1)	981 (31.6)	1138 (36.6)	
Rural	4097 (65.9)	2127 (68.4)	1970 (63.4)	
Education, n (%)				< 0.001
Illiteracy	2935 (47.2)	1364 (43.9)	1571 (50.5)	
Primary school	1357 (21.8)	686 (22.1)	671 (21.6)	
Middle school	1263 (20.3)	674 (21.7)	589 (19)	
High school and above	659 (10.6)	382 (12.3)	277 (8.9)	
Drink, n (%)				< 0.001
no	3724 (59.9)	1734 (55.8)	1990 (64.1)	

yes	2490 (40.1)	1374 (44.2)	1116 (35.9)	
Smoke, n (%)				< 0.001
no	3894 (62.6)	1837 (59.1)	2057 (66.2)	
yes	2322 (37.4)	1271 (40.9)	1051 (33.8)	
Hypertension, n (%)				< 0.001
no	4785 (77.3)	2544 (82.3)	2241 (72.3)	
yes	1407 (22.7)	549 (17.7)	858 (27.7)	
Heart disease, n (%)				< 0.001
no	5523 (89.2)	2836 (91.7)	2687 (86.7)	
yes	669 (10.8)	258 (8.3)	411 (13.3)	
Stroke, n (%)				0.063
no	6084 (98.1)	3052 (98.4)	3032 (97.8)	
yes	118 (1.9)	49 (1.6)	69 (2.2)	
SBP(mmHg)	127.53 ± 20.63	124.77 ± 19.96	130.30 ± 20.92	< 0.001
DBP(mmHg)	74.82 ± 12.07	73.74 ± 12.03	75.91 ± 12.00	< 0.001
Height(m)	1.58 ± 0.08	1.59 ± 0.08	1.57 ± 0.09	< 0.001
Weight(kg)	58.29 ± 10.74	57.65 ± 10.03	58.94 ± 11.38	< 0.001
WC(cm)	84.58 ± 9.80	80.50 ± 8.66	88.65 ± 9.15	< 0.001
BMI(kg/m^2)	23.31 ± 3.51	22.85 ± 3.31	23.76 ± 3.64	< 0.001
WBC($\times10^9$/L)	6.07 ± 1.64	5.92 ± 1.61	6.23 ± 1.66	< 0.001
BUN(mg/dl)	15.58 ± 4.37	15.80 ± 4.49	15.36 ± 4.23	< 0.001
Cr(mg/dl)	0.77 ± 0.18	0.77 ± 0.18	0.77 ± 0.18	0.593
UA(mg/dl)	4.38 ± 1.19	4.28 ± 1.14	4.48 ± 1.23	< 0.001
TC(mg/dl)	192.83 ± 36.53	185.65 ± 34.15	200.01 ± 37.42	< 0.001
TG(mg/dl)	119.32 ± 67.18	84.12 ± 34.07	154.53 ± 73.39	< 0.001
HDL(mg/dl)	52.33 ± 14.89	56.64 ± 14.75	48.02 ± 13.73	< 0.001
LDL(mg/dl)	117.77 ± 33.05	114.29 ± 30.73	121.25 ± 34.88	< 0.001
non-HDL(mg/dl)	140.50 ± 36.02	129.02 ± 32.32	151.99 ± 35.88	< 0.001
HbA1c(%)	5.10 ± 0.39	5.07 ± 0.38	5.13 ± 0.40	< 0.001
FBG(mg/dl)	100.03 ± 11.25	97.35 ± 11.08	102.70 ± 10.77	< 0.001
hsCRP(mg/l)	0.90 (0.51, 1.76)	0.76 (0.46, 1.52)	1.06 (0.59, 1.99)	< 0.001
log(hsCRP)	-0.11 (-0.67, 0.57)	-0.27 (-0.78, 0.42)	0.06 (-0.53, 0.69)	< 0.001
TyG-ABSI	70.88 ± 6.87	65.54 ± 3.84	76.22 ± 4.75	< 0.001
WBC-Z score	-0.11 (-0.72, 0.58)	-0.23 (-0.84, 0.50)	-0.04 (-0.65, 0.69)	< 0.001
log(hsCRP)-Z score	-0.10 (-0.74, 0.67)	-0.29 (-0.87, 0.50)	0.09 (-0.58, 0.81)	< 0.001
Z-score	-0.14 (-1.12, 1.02)	-0.40 (-1.34, 0.77)	0.12 (-0.87, 1.19)	< 0.001

Abbreviations: TyG-ABSI, triglyceride- glucose A Body Shape Index; SBP, systolic blood pressure; DBP, diastolic blood pressure; WC, waist circumference; BMI, body mass index; WBC, white blood cell count; BUN, blood urea nitrogen; Cr, creatinine; UA, uric acid; TC, total cholesterol; TG, triglyceride; HDL, high-density lipoprotein cholesterol; LDL, low-density lipoprotein cholesterol;non-HDL-C,non-high-density lipoprotein cholesterol;HbA1c, glycated hemoglobin; FBG, fasting blood glucose; hsCRP, high-sensitivity C-reactive protein. **Note**: Data are presented as mean ± standard deviation (SD) for normally distributed continuous variables, median (interquartile range, IQR) for non-normally distributed continuous variables, and n (%) for

categorical variables. Z-score was calculated as the sum of WBC-Z score and log(hsCRP)-Z score.

Table S6 Baseline characteristics of participants stratified by low and high total inflammation Z-score levels

Variables	Total (n = 6216)	Low Z-socre (n = 3109)	High Z-socre (n = 3107)	*P*
Age(years)	58.30 ± 8.68	57.81 ± 8.64	58.79 ± 8.70	< 0.001
Gender, n (%)				< 0.001
Male	3452 (55.5)	1821 (58.6)	1631 (52.5)	
Female	2764 (44.5)	1288 (41.4)	1476 (47.5)	
Marital, n (%)				0.196
Single	651 (10.5)	310 (10)	341 (11)	
Married	5565 (89.5)	2799 (90)	2766 (89)	
Location, n (%)				0.016
Urban	2119 (34.1)	1015 (32.6)	1104 (35.5)	
Rural	4097 (65.9)	2094 (67.4)	2003 (64.5)	
Education, n (%)				0.382
Illiteracy	2935 (47.2)	1492 (48)	1443 (46.5)	
Primary school	1357 (21.8)	681 (21.9)	676 (21.8)	
Middle school	1263 (20.3)	605 (19.5)	658 (21.2)	
High school and above	659 (10.6)	330 (10.6)	329 (10.6)	
Drink, n (%)				0.156
no	3724 (59.9)	1890 (60.8)	1834 (59)	
yes	2490 (40.1)	1218 (39.2)	1272 (41)	
Smoke, n (%)				< 0.001
no	3894 (62.6)	2086 (67.1)	1808 (58.2)	
yes	2322 (37.4)	1023 (32.9)	1299 (41.8)	
Hypertension, n (%)				< 0.001
no	4785 (77.3)	2511 (81.1)	2274 (73.5)	
yes	1407 (22.7)	586 (18.9)	821 (26.5)	
Heart disease, n (%)				0.01
no	5523 (89.2)	2794 (90.2)	2729 (88.2)	
yes	669 (10.8)	303 (9.8)	366 (11.8)	
Stroke, n (%)				0.003
no	6084 (98.1)	3061 (98.6)	3023 (97.6)	
yes	118 (1.9)	43 (1.4)	75 (2.4)	
SBP(mmHg)	127.53 ± 20.63	125.24 ± 20.00	129.84 ± 21.00	< 0.001
DBP(mmHg)	74.82 ± 12.07	73.64 ± 11.97	76.00 ± 12.05	< 0.001
Height(m)	1.58 ± 0.08	1.58 ± 0.08	1.58 ± 0.09	0.631
Weight(kg)	58.29 ± 10.74	56.95 ± 10.22	59.63 ± 11.08	< 0.001
WC(cm)	84.58 ± 9.80	82.86 ± 9.13	86.29 ± 10.14	< 0.001
BMI(kg/m^2)	23.31 ± 3.51	22.78 ± 3.25	23.83 ± 3.67	< 0.001
WBC(×10^9/L)	6.07 ± 1.64	5.08 ± 1.04	7.07 ± 1.52	< 0.001
BUN(mg/dl)	15.58 ± 4.37	15.58 ± 4.31	15.58 ± 4.42	0.99

Cr(mg/dl)	0.77 ± 0.18	0.76 ± 0.17	0.79 ± 0.18	< 0.001
UA(mg/dl)	4.38 ± 1.19	4.17 ± 1.13	4.60 ± 1.22	< 0.001
TC(mg/dl)	192.83 ± 36.53	189.21 ± 35.78	196.46 ± 36.93	< 0.001
TG(mg/dl)	119.32 ± 67.18	109.12 ± 59.36	129.54 ± 72.76	< 0.001
HDL(mg/dl)	52.33 ± 14.89	54.22 ± 14.80	50.43 ± 14.74	< 0.001
LDL(mg/dl)	117.77 ± 33.05	115.18 ± 32.32	120.36 ± 33.58	< 0.001
non-HDL(mg/dl)	140.50 ± 36.02	134.99 ± 34.79	146.03 ± 36.40	< 0.001
HbA1c(%)	5.10 ± 0.39	5.06 ± 0.38	5.14 ± 0.40	< 0.001
FBG(mg/dl)	100.03 ± 11.25	99.05 ± 10.87	101.00 ± 11.54	< 0.001
hsCRP(mg/l)	0.90 (0.51, 1.76)	0.55 (0.38, 0.82)	1.69 (0.99, 2.92)	< 0.001
log(hsCRP)	-0.11 (-0.67, 0.57)	-0.60 (-0.97, -0.20)	0.52 (-0.01, 1.07)	< 0.001
TyG-ABSI	70.88 ± 6.87	69.79 ± 6.63	71.98 ± 6.93	< 0.001
WBC-Z score	-0.11 (-0.72, 0.58)	-0.64 (-1.08, -0.17)	0.50 (-0.04, 1.18)	< 0.001
log(hsCRP)-Z score	-0.10 (-0.74, 0.67)	-0.66 (-1.08, -0.20)	0.62 (0.01, 1.24)	< 0.001
Z-score	-0.14 (-1.12, 1.02)	-1.12 (-1.76, -0.61)	1.03 (0.41, 1.83)	< 0.001

Abbreviations: TyG-ABSI, triglyceride- glucose A Body Shape Index; SBP, systolic blood pressure; DBP, diastolic blood pressure; WC, waist circumference; BMI, body mass index; WBC, white blood cell count; BUN, blood urea nitrogen; Cr, creatinine; UA, uric acid; TC, total cholesterol; TG, triglyceride; HDL, high-density lipoprotein cholesterol; LDL, low-density lipoprotein cholesterol;non-HDL-C,non-high-density lipoprotein cholesterol;HbA1c, glycated hemoglobin; FBG, fasting blood glucose; hsCRP, high-sensitivity C-reactive protein. **Note**: Data are presented as mean ± standard deviation (SD) for normally distributed continuous variables, median (interquartile range, IQR) for non-normally distributed continuous variables, and n (%) for categorical variables. Z-score was calculated as the sum of WBC-Z score and log(hsCRP)-Z score.

TableS7 Dose-response relationship of TyG-ABSI and Z-score with incident T2DM by tertiles and quartiles

**Variable**	**Categories**	**HR (95%CI)**	***P***
**TyG-ABSI**			
Tertiles	T1(lowest)	Ref	
	T2	1.29 (1.01~1.64)	0.040
	T3	1.55 (1.21~1.99)	0.001
Quartiles			
	Q1(lowest)	Ref	
	Q2	1.29 (0.97~1.73)	0.083
	Q3	1.58 (1.18~2.1)	0.002
	Q4	1.77 (1.32~2.38)	<0.001
**Z-score**			
Tertiles	T1(lowest)	Ref	
	T2	1.17 (0.93~1.47)	0.170
	T3	1.43 (1.14~1.78)	0.002
Quartiles	Q1(lowest)	Ref	
	Q2	1.13 (0.86~1.47)	0.380
	Q3	1.17 (0.9~1.52)	0.242

Q4	1.44 (1.12~1.85)	0.005

Abbreviations: TyG-ABSI, triglyceride- glucose A Body Shape Index; Z-score was calculated as the sum of WBC-Z score and log(hsCRP)-Z score; HR, hazard ratio; CI, confidence interval. Note: The model was further adjusted for age, gender, smoking status, hypertension, heart disease, stroke, systolic blood pressure (SBP), uric acid (UA), high-density lipoprotein cholesterol (HDL), non-high-density lipoprotein cholesterol (non-HDL-C), and glycated hemoglobin (HbA1c) (the same covariates as primary Model 3).

Table S8 Sensitivity analyses of the joint effects of TyG- ABSI and Z- score on the risk of incident diabetes after excluding participants who developed diabetes within the first 2 years of follow-up (N=6099)

Variable	Model 1		Model 2		Model 3	
	HR (95%CI)	*P*	HR (95%CI)	*P*	HR (95%CI)	*P*
TyG-ABSI						
Low TyG-ABSI	Ref		Ref		Ref	
High TyG-ABSI	1.82 (1.5~2.21)	<0.001	1.69 (1.39~2.07)	<0.001	1.31 (1.05~1.63)	0.015
Z-score						
Low Z-score	Ref		Ref		Ref	
High Z-score	1.56 (1.29~1.89)	<0.001	1.49 (1.23~1.8)	<0.001	1.26 (1.03~1.54)	0.022
TyG-ABSI & Z-score						
Low TyG-ABSI & Low Z-score	Ref		Ref		Ref	
Low TyG-ABSI & High Z-score	1.58 (1.16~2.16)	0.004	1.53 (1.12~2.09)	0.008	1.38 (1.01~1.89)	0.044
High TyG-ABSI & Low Z-score	1.87 (1.38~2.52)	<0.001	1.76 (1.29~2.38)	<0.001	1.42 (1.04~1.95)	0.027
High TyG-ABSI & High Z-score	2.6 (1.98~3.41)	<0.001	2.35 (1.78~3.1)	<0.001	1.67 (1.23~2.25)	0.001

Abbreviations: TyG-ABSI, triglyceride- glucose A Body Shape Index;HR,hazard ratio.**Note**:Z-score was calculated as the sum of WBC-Z score and log(hsCRP)-Z score.

Table S9 Sensitivity analyses of the joint effects of TyG- ABSI and Z- score on the risk of incident diabetes after excluding participants with non- fasting blood samples at baseline (N=5801)

Variable	Model 1		Model 2		Model 3	
	HR (95%CI)	*P*	HR (95%CI)	*P*	HR (95%CI)	*P*
TyG-ABSI						
Low TyG-ABSI	Ref		Ref		Ref	
High TyG-ABSI	1.97 (1.65~2.36)	<0.001	1.83 (1.52~2.2)	<0.001	1.47 (1.2~1.8)	<0.001
Z-score						
Low Z-score	Ref		Ref		Ref	
High Z-score	1.45 (1.22~1.72)	<0.001	1.39 (1.16~1.65)	<0.001	1.18 (0.98~1.41)	0.077
TyG-ABSI & Z-score						
Low TyG-ABSI & Low Z-score	Ref		Ref		Ref	
Low TyG-ABSI & High Z-score	1.31 (0.98~1.75)	0.071	1.28 (0.96~1.71)	0.097	1.17 (0.87~1.57)	0.294
High TyG-ABSI & Low Z-score	1.87 (1.43~2.44)	<0.001	1.75 (1.33~2.3)	<0.001	1.47 (1.11~1.95)	0.007
High TyG-ABSI & High Z-score	2.52 (1.97~3.21)	<0.001	2.29 (1.79~2.94)	<0.001	1.69 (1.29~2.22)	<0.001

Abbreviations: TyG-ABSI, triglyceride- glucose A Body Shape Index;HR,hazard ratio.Note:Z-score was calculated as the sum of WBC-Z score and log(hsCRP)-Z score.

Table S10 Sensitivity analyses for the association of TyG, ABSI, Z score and their combination with incident T2DM under different adjustment strategies

**Variable**	**Model A** HR(95CI%)	***P***	**Model B** HR(95CI%)	***P***	**Model C** HR(95CI%)	***P***
TyG-ABSI						
Low TyG-ABSI	Ref		Ref		Ref	
High TyG-ABSI	1.46 (1.2~1.77)	<0.001	1.42 (1.17~1.73)	<0.001	1.46 (1.21~1.78)	<0.001
Z-score						
Low Z-score	Ref		Ref		Ref	
High Z-score	1.28 (1.08~1.53)	0.005	1.21 (1.02~1.45)	0.032	1.29 (1.08~1.53)	0.005
TyG-ABSI & Z-score						
Low TyG-ABSI & Low Z-score	Ref		Ref		Ref	
TyG-ABSI	1.29 (0.97~1.71)	0.079	1.24 (0.93~1.64)	0.140	1.29 (0.98~1.72)	0.074
Low TyG-ABSI	1.46 (1.11~1.93)	0.007	1.45 (1.1~1.92)	0.008	1.48 (1.12~1.94)	0.006
High TyG-ABSI	1.83 (1.41~2.39)	<0.001	1.7 (1.3~2.22)	<0.001	1.85 (1.42~2.41)	<0.001

Abbreviations: TyG-ABSI, triglyceride-glucose A Body Shape Index; Z-score was calculated as the sum of WBC-Z score and log(hsCRP)-Z score; HR, hazard ratio; CI, confidence interval. **Note:** All models included the identical covariate set as the primary Model 3 (age, gender, smoking status, hypertension, heart disease, stroke, systolic blood pressure (SBP), uric acid (UA), high-density lipoprotein cholesterol (HDL), non-high-density lipoprotein cholesterol (non-HDL-C), and glycated hemoglobin (HbA1c)) except for the following changes. Model A excluded HbA1c. Model B additionally adjusted for lipid-lowering medication use (yes/no). Model C both excluded HbA1c and adjusted for lipid-lowering medication use.

Table S11 Comparison of primary and sensitivity analyses for the joint association of TyG- ABSI and Z- score with incident T2DM

Joint exposure group	Primary analysis (MICE + standard Cox) HR (95% CI)	Sensitivity analysis (CCA + survey- weighted Cox) HR (95% CI)
Low TyG-ABSI & Low Z-score	Ref	Ref
Low TyG-ABSI & High Z-score	1.23 (0.93–1.64)	1.38 (1.00–1.90)
High TyG-ABSI & Low Z-score	1.44 (1.09–1.90)	1.55 (1.10–2.19)
High TyG-ABSI & High Z-score	1.69 (1.30–2.21)	1.90 (1.38–2.61)

Abbreviations: TyG-ABSI, triglyceride-glucose A Body Shape Index; Z-score was calculated as the sum of WBC-Z score and log(hsCRP)-Z score; MICE, multiple imputation by chained equations; CCA, complete case analysis;.HR, hazard ratio; CI, confidence interval. **Note:** Primary analysis used multiple imputation (MICE) for missing covariates and standard Cox regression. Sensitivity analysis used complete case analysis (excluding 155 participants with missing data) and survey- weighted Cox regression to account for the complex sampling design of CHARLS. All models were adjusted for the same covariates as Model 3 (age, sex, smoking, hypertension, heart disease, stroke, SBP, UA, HDL, non- HDL- C, and HbA1c).

Table S12 Additive and multiplicative interactions between TyG- ABSI and Z- score in relation to the risk of incident diabetes

	Additive effect			Multiplicative effect
	RERI	AP	SI	
Interactive effects (95% CI)	0.02(-0.44-0.47)	0.01(-0.26-0.28)	1.03(0.52-2.02)	0.95(0.67-1.35)

Abbreviations: TyG-ABSI, triglyceride-glucose A Body Shape Index; RERI, relative excess risk due to interaction; AP, attributable proportion due to interaction; SI, synergy index; CI, confidence interval.

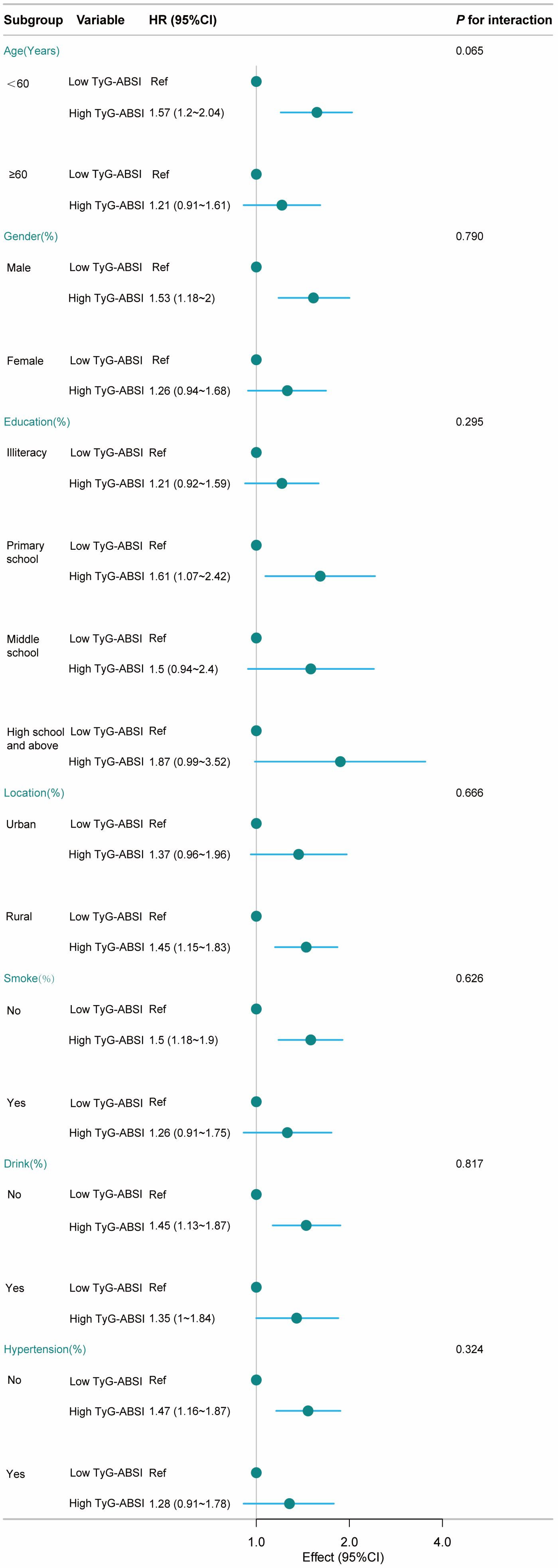

Fig. S1 Subgroup analysis of the association between TyG-ABSI levels and incident diabetes risk.
Abbreviations: TyG-ABSI, triglyceride-glucose A Body Shape Index.

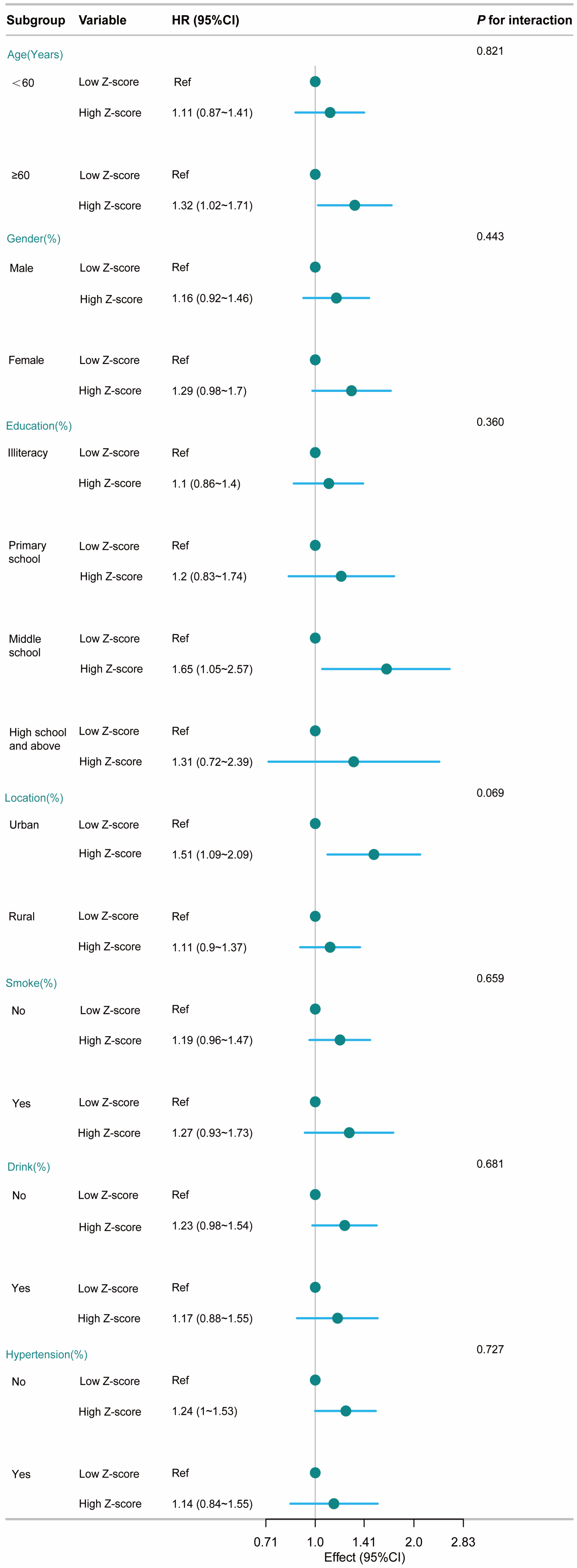

Fig. S2 Subgroup analysis of the association between Z-score levels and incident diabetes risk. **Note:** The Z? score was calculated as the sum of the white blood cell count Z-score and the log(hsCRP) Z-score.

A

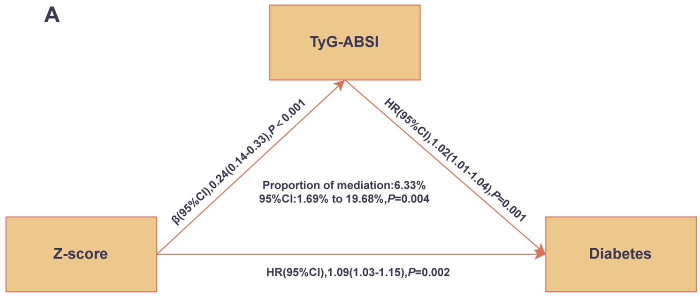

B

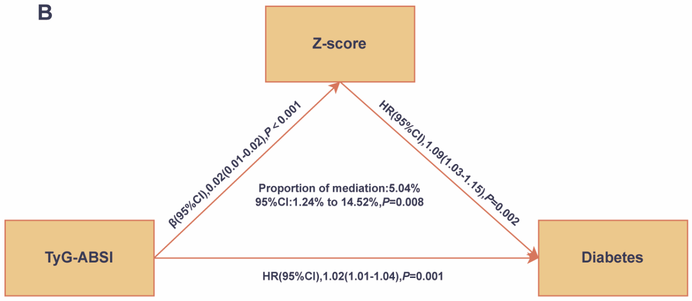

Fig.S3 Bidirectional mediation analysis of the association between TyG-ABSI, Z-score, and incident diabetes. (A) Mediation effect of TyG-ABSI on the association between Z-score and incident diabetes. (B) Mediation effect of Z-score on the association between TyG?ABSI and incident diabetes. **Abbreviations**: TyG-ABSI, triglyceride-glucose A Body Shape Index.**Note:** The Z-score was calculated as the sum of the white blood cell count Z-score and the log(hsCRP) Z-score. The percentages indicate the proportion of total effect mediated (with 95% confidence interval), calculated as (indirect effect / total effect) × 100%. *P* values are from bootstrap tests for the indirect effect
